# Enhanced Interleukin-1 Activity Contributes to Exercise Intolerance in Patients with Systolic Heart Failure

**DOI:** 10.1371/journal.pone.0033438

**Published:** 2012-03-16

**Authors:** Benjamin W. Van Tassell, Ross A. Arena, Stefano Toldo, Eleonora Mezzaroma, Tania Azam, Ignacio M. Seropian, Keyur Shah, Justin Canada, Norbert F. Voelkel, Charles A. Dinarello, Antonio Abbate

**Affiliations:** 1 School of Pharmacy, Virginia Commonwealth University, Richmond, Virginia, United States of America; 2 Victoria Johnson Center, Virginia Commonwealth University, Richmond, Virginia, United States of America; 3 Physical Therapy Program, University of New Mexico, Albuquerque, New Mexico, United States of America; 4 Virginia Commonwealth University Pauley Heart Center, Virginia Commonwealth University, Richmond, Virginia, United States of America; 5 Department of Internal Medicine, University of Colorado, Boulder, Colorado, United States of America; 6 Department of Physical Therapy, Virginia Commonwealth University, Richmond, Virginia, United States of America; National Institutes of Health, United States of America

## Abstract

**Background:**

Heart failure (HF) is a complex clinical syndrome characterized by impaired cardiac function and poor exercise tolerance. Enhanced inflammation is associated with worsening outcomes in HF patients and may play a direct role in disease progression. Interleukin-1β (IL-1β) is a pro-inflammatory cytokine that becomes chronically elevated in HF and exerts putative negative inotropic effects.

**Methods and Results:**

We developed a model of IL-1β-induced left ventricular (LV) dysfunction in healthy mice that exhibited a 32% reduction in LV fractional shortening (P<0.001) and a 76% reduction in isoproterenol response (P<0.01) at 4 hours following a single dose of IL-1β 3 mcg/kg. This phenotype was reproducible in mice injected with plasma from HF patients and fully preventable by pretreatment with IL-1 receptor antagonist (anakinra). This led to the design and conduct of a pilot clinical to test the effect of anakinra on cardiopulmonary exercise performance in patients with HF and evidence of elevated inflammatory signaling (n = 7). The median peak oxygen consumption (VO_2_) improved from 12.3 [10.0, 15.2] to 15.1 [13.7, 19.3] mL·kg^–1^·min^–1^ (P = 0.016 vs. baseline) and median ventilator efficiency (V_E_/VCO_2_ slope) improved from 28.1 [22.8, 31.7] to 24.9 [22.9, 28.3] (P = 0.031 vs. baseline).

**Conclusions:**

These findings suggest that IL-1β activity contributes to poor exercise tolerance in patients with systolic HF and identifies IL-1β blockade as a novel strategy for pharmacologic intervention.

**Trial Registration:**

ClinicalTrials.gov NCT01300650

## Introduction

Heart failure (HF) is a complex clinical syndrome characterized by dyspnea, fatigue, and poor exercise tolerance. [1]–[2] While contemporary HF treatments have slowed disease progression and improved survival, the overall incidences of HF morbidity and mortality continue to rise, suggesting that the current treatment paradigm still misses one or more key pathophysiologic mechanisms. [1]–[2]

Among HF patients, a significant correlation exists between declining functional class and increasing levels of inflammatory cytokines. [3]–[4] Interleukin-1β (IL–1β) is the prototypal inflammatory cytokine that acts as an acute phase reactant following tissue injury (i.e. ischemia) and becomes persistently elevated in patients with chronic HF. [5]–[7] Early investigations in septic cardiomyopathy identified IL-1β as a soluble ‘depressant factor’ in the sera of these patients, producing a concentration-dependent depression of myocyte contractility *in vitro* [7] Further studies identify a pathologic role for IL-1β in ventricular remodeling, systolic dysfunction, and cardiomyocyte death in both ischemic and non-ischemic models of HF. [7]–[12] IL-1β has also been shown to affect β-adrenergic receptor responsiveness *in vitro*, which may be a key determinant of exercise capacity in HF. [13]–[14] Nevertheless, the precise contribution of IL-1β to human HF has not been well established.

We therefore designed a bench-to-bedside approach to determine the contribution of IL-1β in the development of cardiac dysfunction and ultimately guide the use of IL-1β blockade in a pilot study of patients with HF. We first conducted a dose ranging study with IL-1β in healthy mice to induce LV dysfunction at rest and impaired responsiveness to β-adrenergic receptor (β-AR) stimulation. We then observed that mice injected with plasma from HF patients exhibited a similar phenotype of impaired systolic dysfunction that was completely prevented by pre-treatment with recombinant IL-1 receptor antagonist (IL-1Ra). These observations led to the current pilot study in human patients with HF in which 2 weeks treatment with anakinra improved cardiopulmonary exercise performance as measured by peak oxygen consumption, ventilatory efficiency, and exercise time.

## Methods

The protocol for this trial is available as supporting information; see Protocol S1.

### Mouse model of IL-1β-induced systolic dysfunction

#### Animals

Adult outbreed Institute of Cancer Research (ICR) male mice were purchased from Harlan Sprague-Dawley (Indianapolis, IN). Mice were 30 – 35 g at the time of the study. All protocols involving animals were reviewed and approved by the Animal Care and Use Committee of Virginia Commonwealth University. Each group included between 6–10 mice.

#### Dose response

Mice received intraperitoneal (IP) injection with recombinant human IL-1β (Cell Sciences, Canton, MA) with transthoracic echocardiography to measure left ventricular (LV) fractional shortening at baseline, 4 hours, and 24 hours. IL-1β was administered at doses of 1 ng, 10 ng, 100 ng, and 1000 ng (0.03 mcg/kg, 0.3 mcg/kg, 3 mcg/kg, and 30 mcg/kg) diluted in 0.2 mL of normal saline (NaCl 0.9%).

#### Echocardiography

Mice underwent transthoracic echocardiography under light anesthesia (pentobarbital 30–50 mg/kg IP). The chest was shaved and the mice were placed supine on a heating pad. Doppler echocardiography was performed with the Vevo770 imaging system (VisualSonics Inc, Toronto, Ontario, Canada) and a 30-MHz probe. The transducer was positioned on the left anterior side of the chest. The heart was first imaged in the 2-dimensional mode in the short-axis view of the left ventricle. Then, M-mode images were obtained at the level of the papillary muscles below the mitral valve tip according to the American Society of Echocardiography recommendations. [15] The M-mode cursor was positioned perpendicular to the anterior and posterior wall to measure the left ventricular (LV) end-diastolic diameter (LVEDD) and end-systolic diameter (LVESD). LV fractional shortening (LVFS) was calculated as follows: LVFS = (LVEDD–LVESD)/LVEDDx100. LV ejection fraction (LVEF) was calculated with the Teichholz formula. [15] Transmitral and left ventricle outflow tract pulsed Doppler flow spectra were obtained from the apical view. Measurement of the outflow tract flow was performed and isovolumetric contraction (ICT) and relaxation (IRT) times and ejection time (ET) were measured. LV outflow tract (LVOT) flow velocity–time integral (AoVTI) was also determined, with LVOT measured as the cross-sectional area in the parasternal long-axis view. These data were used to calculate the Tei index (Tei index = ICT+IRT/ET). In humans, a higher Tei index is associated with both systolic and diastolic dysfunction and worse outcomes. [15] Velocity of circumferential shortening was calculated as FS/ET, with ET divided by the square root of the preceding RR interval to correct for heart rate. [16] All echocardiograms were performed and interpreted jointly by 2 investigators (BVT and AA) throughout all experiments.

#### Contractile reserve

Mice received a single IP injection of a β adrenergic receptor agonist (isoproterenol, 0.01 mcg/kg; SigmaAldrich, St. Louis, MO) to calculate contractile reserve. Contractile reserve was defined as the maximum percent increase in LVFS within 5 minutes after isoproterenol.

### Effects of plasma from patients with HF injected into healthy mice

We collected blood from four groups of patients based upon HF symptoms and elevation of high sensitivity C-reactive protein (hsCRP, a surrogate marker of IL-1β activity): healthy controls (CTRL), ambulatory HF patients with low CRP (HF L-CRP), ambulatory HF with high CRP (HF H-CRP), and patients hospitalized for acute decompensated HF (ADHF). For inclusion in this stage of the investigation, healthy controls were defined as patients with no history of cardiac disease. Ambulatory HF patients were defined as patients with a documented history of HF with LV ejection fraction (LVEF) <40% but without any recent hospitalizations (previous 12 months) or recent changes in medications (previous 3 months). Patients with acute decompensated HF were eligible if they had been admitted with a confirmed diagnosis of acute decompensated HF within the previous 24 hours. Plasma samples from each group were injected into healthy mice who underwent transthoracic echocardiography to measure LV function at baseline and 4 hours. To neutralize the effects of IL-1β on LV function, separate groups of mice received injections of the same human plasma following pre-treatment with recombinant human IL-1 receptor antagonist (anakinra, Kineret®, Biovitrum) 100 mg/kg IP administered 30 minutes prior to injection of human plasma. Upon completion of echocardiography at 4 hours, all groups of mice received an injection of isoproterenol 0.01 mcg/kg IP to evaluate contractile reserve.

### Pilot study of IL-1β blockade in patients with stable HF

#### Study design

We conducted an open-label, single-arm, pilot-study to evaluate the safety and feasibility of IL-1β blockade to improve aerobic exercise performance in ambulatory patients with HF and high hsCRP. The study was registered on ClinicalTrials.gov (NCT01300650) and received an exemption for investigational new drug use from the Food and Drug Administration according to current federal regulations (Code of Federal Regulations, 312.2[b]). All human subjects research was conducted at Virginia Commonwealth University. The study design and protocol received approval from the VCU Institutional Review Board and all patients provided written informed consent. Patients presenting to the VCU Cardiology Clinic were screened for potential enrollment. Following enrollment, patients completed a HF symptom questionnaire (Duke Activity Status Index [DASI], see Appendix) [17] and underwent baseline cardiopulmonary exercise testing (CPX). Patients then received a 14-day supply of anakinra (100 mg subcutaneous injection daily) accompanied by detailed instructions on drug administration, dosing, and storage. Patients were then scheduled for a repeat questionnaire and CPX upon completion of the anakinra regimen.

#### Entry criteria

The inclusion criteria were age >18 years, a diagnosis of HF, documented LVEF <40%, and hsCRP >2 mg/L. The exclusion criteria were recent changes (previous 3 months) in HF maintenance medications (beta-blockers, angiotensin converting enzyme [ACE] inhibitors, aldosterone antagonists, vasodilators, digoxin, diuretics); hospitalization for worsening HF or acute decompensated HF within the previous 12 months; anticipated need for cardiac resynchronization therapy (CRT) or automated-implantable cardioverter defibrillator (AICD); angina or electrocardiograph (ECG) changes that limit maximum exertion during CPX or baseline ECG changes that limit the ability to detect ischemia (i.e. left bundle-branch block); recent (<14 days) use of anti-inflammatory drugs (not including NSAIDs), chronic inflammatory disorder (including but not limited to rheumatoid arthritis, systemic lupus erythematosus), malignancy, active infection, or any comorbidity limiting survival or ability to complete the study; severe kidney dysfunction (eGFR <30 mL/min); coagulopathy (INR >1.5), thrombocytopenia (<50,000/mm^3^), or leukopenia (absolute neutrophil count <1,500/mm^3^); pregnancy (female patients were required to take a urine pregnancy test); latex or rubber allergy; or inability to give informed consent.

#### Cardiopulmonary Exercise Testing

A physician-supervised maximal CPX was administered using a metabolic cart that is interfaced with a treadmill (Vmax Encore, Viasys, Yorba Linda, CA). A conservative ramping treadmill protocol was used as described previously. [18] Prior to each test, the oxygen and carbon dioxide sensors were calibrated using gases of known oxygen, nitrogen, and carbon dioxide concentrations and the flow sensor was also calibrated using a 3-Liter syringe. Subjects were briefed regarding the protocol and were requested to exercise to fatigue. 12-lead ECG monitoring were conducted at baseline, throughout the test and into recovery. Blood pressure was measured every two minutes using an automated exercise-compatible device (Tango, SunTech Medical). In this technique, expired gases were sampled using a mouthpiece-mounted sensor, and analyzed to continuously measure oxygen consumption (VO_2_), carbon dioxide production (VCO_2_) and minute ventilation (VE). The highest 10-second average value for VO_2_ during the last 30 seconds of exercise defined the peak value (peak VO_2_ in mL·kg^–1^·min^–1^). Ten second averaged VE and VCO_2_ data, from the initiation of exercise to peak, were input into spreadsheet software (Microsoft Excel, Microsoft Corp., Seattle, WA) to calculate the V_E_/VCO_2_ slope via least squares linear regression (y = mx+b, m = slope). The oxygen uptake efficiency slope (OUES) was determined using least-squares linear regression analysis (oxygen consumption = a log_10_VE+b, with VO_2_ and VE expressed in liters per minute) using spreadsheet software (Microsoft Excel, Microsoft, Seattle, Washington). [19] All exercise data were also used to calculate the OUES. American Heart Association/American College of Cardiology guidelines for exercise testing contraindications and termination criteria were followed. [20]

#### Heart Failure Symptom Questionnaire

The Duke Activity Status Index (DASI) questionnaire is a 12-question, yes/no, instrument that allows for the calculation of perceived functional capacity. [17] Each question describes a different physical activity and asks the subjects if they feel they can perform the task. The questions are weighted according to their degree of physical exertion. The weighted values from the “yes” responses are summed to produce a VO_2_ score (see Appendix). [17]

#### Blood collection

Blood was collected from patients at screening and after anakinra treatment (immediately prior to final CPX) for analysis of hsCRP, brain natriuretic peptide (BNP), complete blood count, and inflammatory cytokines.

#### Cytokine assay

Freshly obtained blood was centrifuged at 2000×*g* for 15 minutes followed by plasma separation and storage at –80°C. Samples were analyzed with the MOSAIC Cytokine Panel 1 (R and D Systems, Minneapolis, MN) using a QuanSys Imager (Logan, UT).

#### Statistical Analysis

Values derived from the experimental animal studies are presented as mean and standard error of mean. The differences between mouse treatment groups were compared using the Student’s T-test for unpaired data when comparing 2 groups or using the ANOVA when comparing 3 or more groups. When comparing changes compared to baseline between 2 experimental groups we used the ANOVA for repeated measures assessing the time_x_group interaction. Data derived from the pilot clinical trial are reported as the median and interquartile range for potential deviation from Gaussian distribution. The differences between baseline and final measurements computed using the Wilcoxon signed-rank test for continuous variables or Fisher’s exact test for discrete variables. Unadjusted p values are reported throughout, with statistical significance set at the 2-tailed 0.05 level. The analyses were completed using the Statistical Package for Social Sciences, version 11.0.1, software (SPSS, Chicago, Illinois).

## Results

### IL-1β i nduces systolic function and impaired contractile reserve

**Figure 1:**
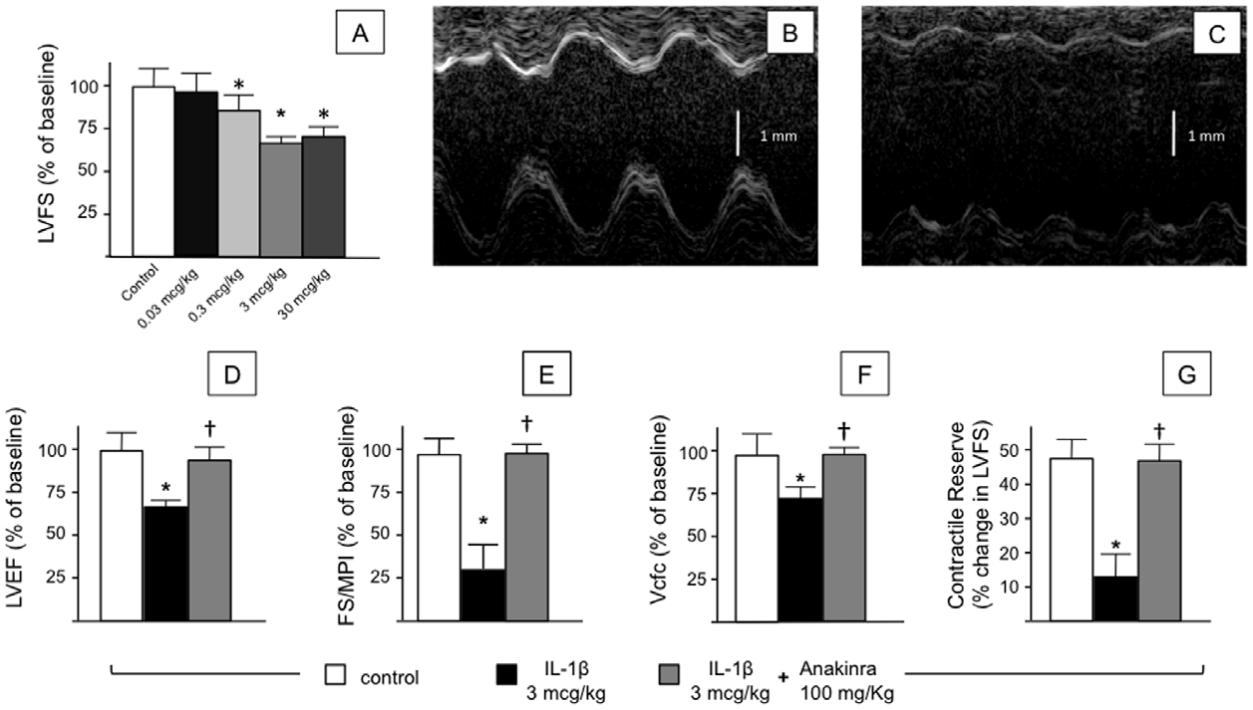
Model of IL-1-induced systolic dysfunction in healthy mice. Healthy, adult, mice underwent baseline echocardiography followed by a single intraperitoneal injection of recombinant human IL-1β (0, 0.03, 0.3, 3, or 30 mcg/kg) and subsequent echocardiography at 4 hours. (A) All doses of IL-1β ≥0.3 mcg/kg produced a significant 28 – 32% reduction in left ventricular fractional shortening (LVFS) at 4 hours. Panels B (baseline) and C (4 hours) show representative echocardiographic images at 4 hours of mice injected with 3 mcg/kg IL-1β. Panels D – F show additional measures of LV function at 4 hours: (D) LV ejection fraction (LVEF); (E) FS/myocardial performance index (MPI) or FS-Tei index; (F) velocity of circumferential fiber shortening (Vcfc) corrected for heart rate. In all experiments, pre-treatment with anakinra 100 mg/kg 30 minutes prior to IL-1β was sufficient to prevent changes in LV function. (G) Isoproterenol 0.01 mcg/kg induced a reproducible 46% increase in LVFS that was significantly blunted by pre-treatment with IL-1β 3 mcg/kg. *P<0.01 versus control/baseline; †P<0.01 versus IL-1β.

To the hypothesis that IL-1β induces systolic dysfunction, we injected healthy mice with increasing doses of IL-1β and measured changes in cardiac function by transthoracic echocardiography. At 4 hours after injection, IL-1β produced significant reductions in LVFS at all doses ≥0.3 mcg/kg (Figure 1A-1C). We further characterized IL-1β 3 mcg/kg as a standard dose in all subsequent experiments as this dose was 10X the minimum dose required to significantly impair contractile function and appeared to give the greatest numerical reduction in LVFS. In addition to changes in contractile indices such as the ratio of fractional shortening/myocardial performance index (FS/MPI) and circumferential shortening–both of which are less sensitive to changes in preload [21], [22]–IL-1β 3 mcg/kg significantly reduced LV stroke volume from 41±2 µL to 29±3 µL (–27%, P<0.001) and increased heart rate from 345±22 beats/min to 432±30 beats/min (+25%, P = 0.004). Estimated cardiac output remained unchanged. No effects were noted with IL-1β doses less than 0.3 mcg/kg. The effects of IL-1β 3 mcg/kg were reproduced by IP administration of IL-1α 3 mcg/kg (data not shown) and prevented by pre-administration of anakinra 100 mg/kg (Figure 1D-1F), suggesting conserved signaling through the IL-1 type 1 membrane receptor (IL-1R_1_).

We then evaluated the effect of IL-1β on contractile reserve in response to a single injection of isoproterenol (0.01 mcg/kg IP). This dose of isoproterenol elicited a 46±5% increase in LVFS in healthy (untreated) mice within 5 minutes of injection, that was significantly blunted when isoproterenol was administered 4 hours after IL-1β administration (11±6% increase, P<0.01 versus untreated mice), revealing a phenotype of impaired response to β-adrenergic receptor agonist (Figure 1G).

### Plasma from HF patients with elevated hsCRP induced LV systolic dysfunction and impaired contractile reserve in healthy mice

**Figure 2:**
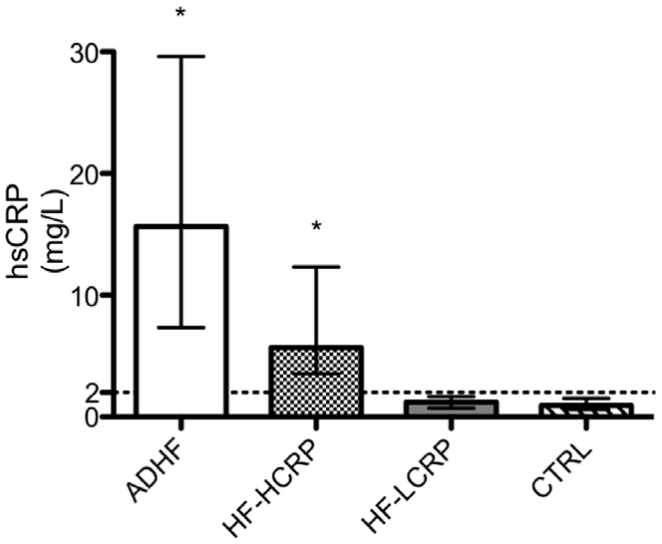
Plasma high sensitivity C-reactive protein (hsCRP). Blood samples were collected from patients from acute decompensated HF (ADHF), HF with high hsCRP (HF-HCRP), HF with low hsCRP (HF-LCRP), and healthy control patients (CTRL) without any cardiovascular disease. High hsCRP was defined as>2 mg/L. *P<0.05 versus CTRL.

**Figure 3:**
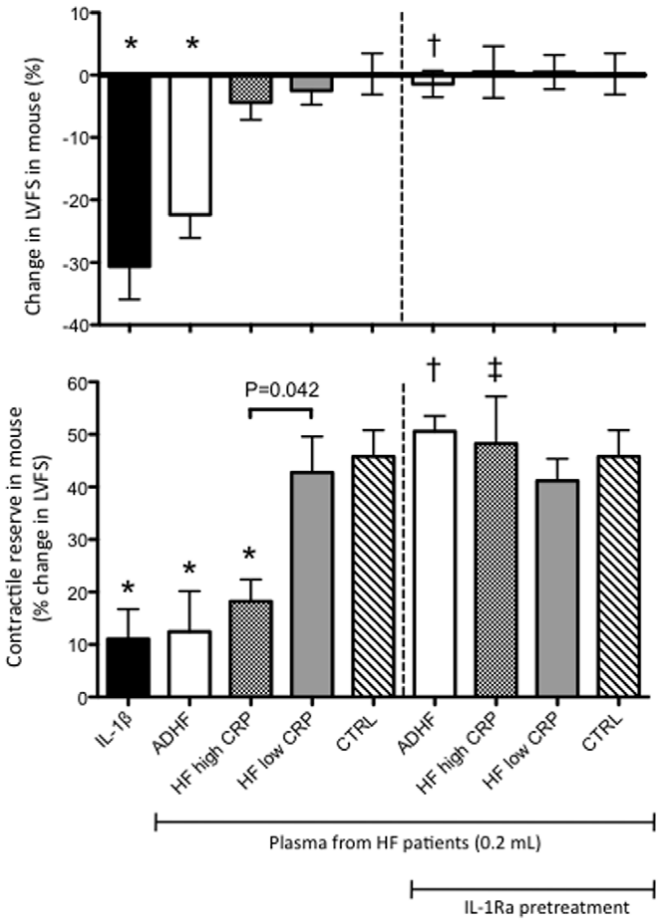
Effects of plasma from HF patients on LV function in healthy mice. Plasma from HF patients (0.2 mL) was injected into healthy mice, follwed by echocardiographic assessment of LV function at 4 hours. Plasma from patients with ADHF induced a 22% reduction in LV fractional shortening (LVFS) in the mouse that was preventable by pretreating the mice with anakinra 100 mg/kg given 30 minutes prior to plasma injection. Plasma from patients with ADHF and HF high CRP reduced contractile reserve in the mouse as measured by LVFS response to isoproterenol. Pre-treating the mice with anakinra 100 mg/kg given 30 minutes prior to plasma injection prevented the reduction in contractile reserve. *P<0.001 versus CTRL, †P<0.05 versus ADHF, ‡P<0.05 versus HF high CRP.

To test whether the plasma from HF patients could reproduce the IL-1β phenotype of cardiac dysfunction, we injected healthy mice with plasma collected blood from four different patient groups: acute decompensated HF (ADHF), stable chronic HF with high hsCRP (HF-HCRP), stable chronic HF with low hsCRP (HF-LCRP), and healthy control (CTRL) patients with no cardiovascular disease. As expected, patients with ADHF and HF-HCRP exhibited higher inflammatory activity as evidenced by elevated hsCRP compared to HF-LCRP and CTRL patients (P<0.05, Figure 2). Of the four patient groups tested, only plasma from ADHF patients induced any significant reduction in resting cardiac function (Figure 3A). However, this dysfunction was completely prevented by pre-treatment with anakinra (P<0.001 vs ADHF), indicating a critical role for IL-1β signaling in the dysfunction (Figure 3, uppler panel). Plasma from patients with HF-HCRP, HF-LCRP, and CTRL patients showed no significant reduction in resting cardiac function.

To test whether HF plasma would also influence contractile reserve, we then injected each mouse with a single dose of isoproterenol (Figure 3, lower panel). Plasma from both patient groups with high inflammatory burden (ADHF and HF-HCRP) blunted the response to isoproterenol, whereas mice injected with plasma from patients without high inflammatory burden (HF-LCRP and CTRL) remained fully sensitive to isoproterenol injection. The blunted response to isoproterenol was completely eliminated by pre-treatment with anakinra, indicating a critical role for IL-1β signaling in the impaired contractile reserve induced by plasma from ADHF and HF-HCRP patients. Among mice injected with plasma form patients with stable chronic HF, there was a significant difference in contractile reserve based upon hsCRP status (HF-HCRP vs HF-LCRP, P = 0.042) that also disappeared with anakinra pre-treatment.

### Pilot study

#### Enrollment

**Figure 4:**
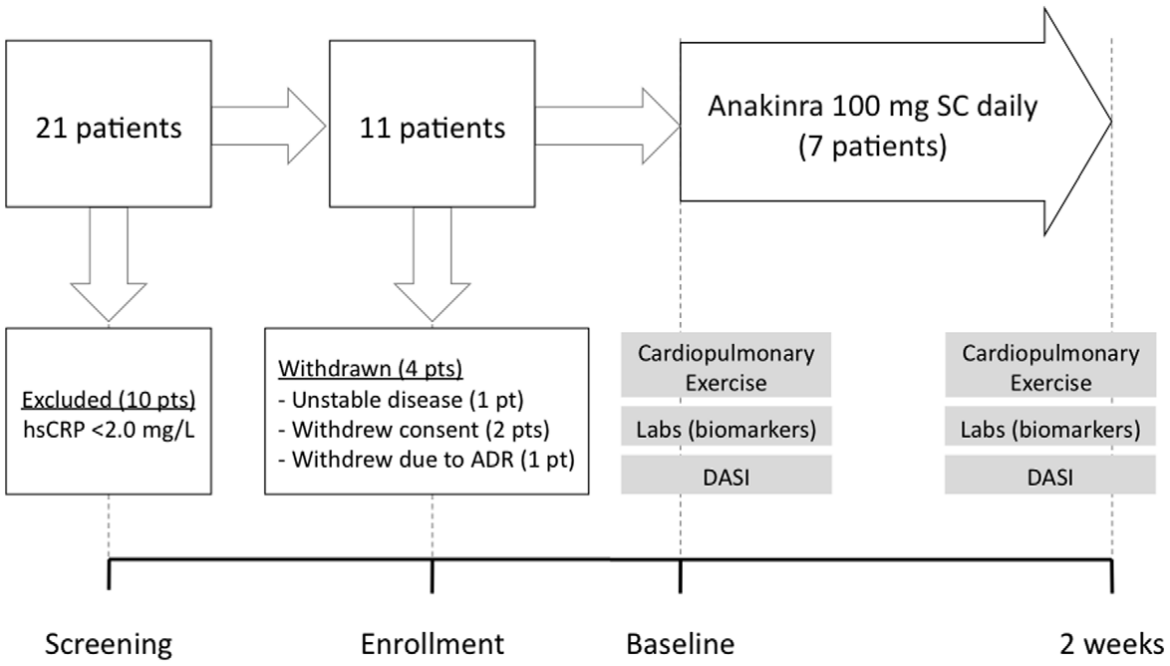
Pilot clinical study. Patients with a diagnosis of stable HF (no hospitalizations within 12 months, no medication changes within 3 months) and documented LV dysfunction (LV ejection fraction <40%) underwent screening for hsCRP >2 mg/L to qualify for a pilot clinical study. Enrolled patients received anakinra 100 mg subcutaneously every day for 2 weeks and the co-primary outcomes were change in peak oxygen consumption (VO_2_) and the slope of minute ventilation over carbon dioxide production (V_E_/VCO_2_). Secondary outcomes included inflammatory biomarkers and the Duke Activity Status Index (DASI), a patient-reported survey of HF symptoms.

Patient screening commenced on February 15, 2011. 19 patients met initial entry criteria and underwent laboratory screening for elevation of hsCRP. A total of 11 patients met full entry criteria and were enrolled in the study. Two patients withdrew consent and another patient was revealed to have unstable HF symptoms prior to baseline exercise testing, leaving 8 patients who underwent baseline CPX and received anakinra injections (Figure 4). Seven patients completed both study visits and 1 patient experienced systemic flu-like symptoms and withdrew from the study after 8 days of treatment. The remaining 7 patients were included in the analysis. Baseline patient characteristics are displayed in Table 1.

**Table 1:** Patient characteristics in pilot clinical study.

	Baseline (n = 7)
**Demographics**	
Age, y	48 [44, 56]
Race, AA, n (%)	7 (100)
Male, n (%)	3 (43)
**Patient Characteristics**	
Height (m)	1.65 [1.59, 1.75]
Weight (kg)	100 [82, 117]
BMI (kg/m^2^)	35.4 [25.9, 42.0]
LV ejection fraction (%)	30 [23], [35]
NYHA function class	2 [2,2]
Estimated GFR (mL/kg/1.73m^2^)	62 [57, 86]
CHF, n (%)	7 (100)
- Ischemic	4 (57)
- Non-ischemic (hypertensive)	3 (43)
Hypertension, n (%)	5 (71)
Diabetes, n (%)	3 (43)
CKD, n (%)	2 (29)
COPD/Asthma, n (%)	1 (14)
**Medications**	
β-AR blocker, n (%)	7 (100)
ACEI/ARB, n (%)	7 (100)
Furosemide, n (%)	6 (86)
Aspirin, n (%)	6 (86)
Statin, n (%)	5 (71)
Spironolactone, n (%)	5 (71)
Nitrates, n (%)	2 (29)
Hydralazine, n (%)	1 (14)

Abbreviations: AA (African American); ACEI (angiotensin converting enzyme inhibitor); ARB (angiotensin receptor blocker); β-AR (β-adrenergic receptor); BMI (body mass index); CAD (coronary heart disease); CHF (chronic heart failure); CKD (chronic kidney disease); COPD (chronic obstructive pulmonary disease); MI (myocardial infarction); NYHA (New York Heart Association).

### IL-1 blockade reduced inflammatory cytokines and biomarkers in patients with HF

Two weeks treatment with anakinra reduced median plasma hsCRP by 84% (5.7 mg/L to 0.9 mg/L, P = 0.016). Absolute neutrophil count underwent a significant reduction, but no patients developed clinically significant neutropenia (ANC <1.8×10^9^ cells/L, Table 2). A subset of patients (n = 3) provided additional plasma for analysis of inflammatory cytokines. While the limited sample size prohibited statistical analysis, median IL-1β and IL-6 concentrations were reduced by 90.0% (12.6 pg/mL to 1.3 pg/mL) and 90% (9.9 pg/mL to 1.0 pg/mL), respectively, while TNFα concentrations appeared unchanged (30.3 pg/mL to 32.1 pg/mL).

**Table 2:** Laboratory values in pilot clinical study.

	Baseline (n = 7)	Final (n = 7)	P
hsCRP, mg/L	5.69 [3.62, 9.48]	0.94 [0.66, 1.77]	**0.016**
BNP, pg/mL	22 [16, 559]	37 [15, 312]	0.30
Platelets×10^9^/L	316 [156, 322]	229 [173, 266]	0.52
Hemoglobin, g/dL	13.7 [12.8, 13.9]	13.1 [12.5, 13.6]	0.73
WBC×10^9^/L	7.3 [6.7, 8.3]	5.4 [4.8, 7.1]	**0.022**
Neutrophils×10^9^/L	4.4 [3.9, 5.3]	2.8 [2.0, 3.7]	**0.016**
Lymphocytes×10^9^/L	2.4 [1.8, 2.6]	2.2 [1.6, 2.6]	0.90
Monocytes×10^9^/L	0.4 [0.4, 0.5]	0.4 [0.3, 0.5]	0.17
Eosinophils×10^9^/L	0.2 [0.2, 0.3]	0.2 [0.2, 0.3]	0.61
Basophils×10^9^/L	0.0 [0.0, 0.05]	0.0 [0.0, 0.0]	0.85

Abbreviations: BNP (brain natriuretic peptide); hsCRP (high sensitivity C-reactive protein); WBC (white blood cell).

### IL-1 blockade improved CPX performance in patients with HF

**Figure 5:**
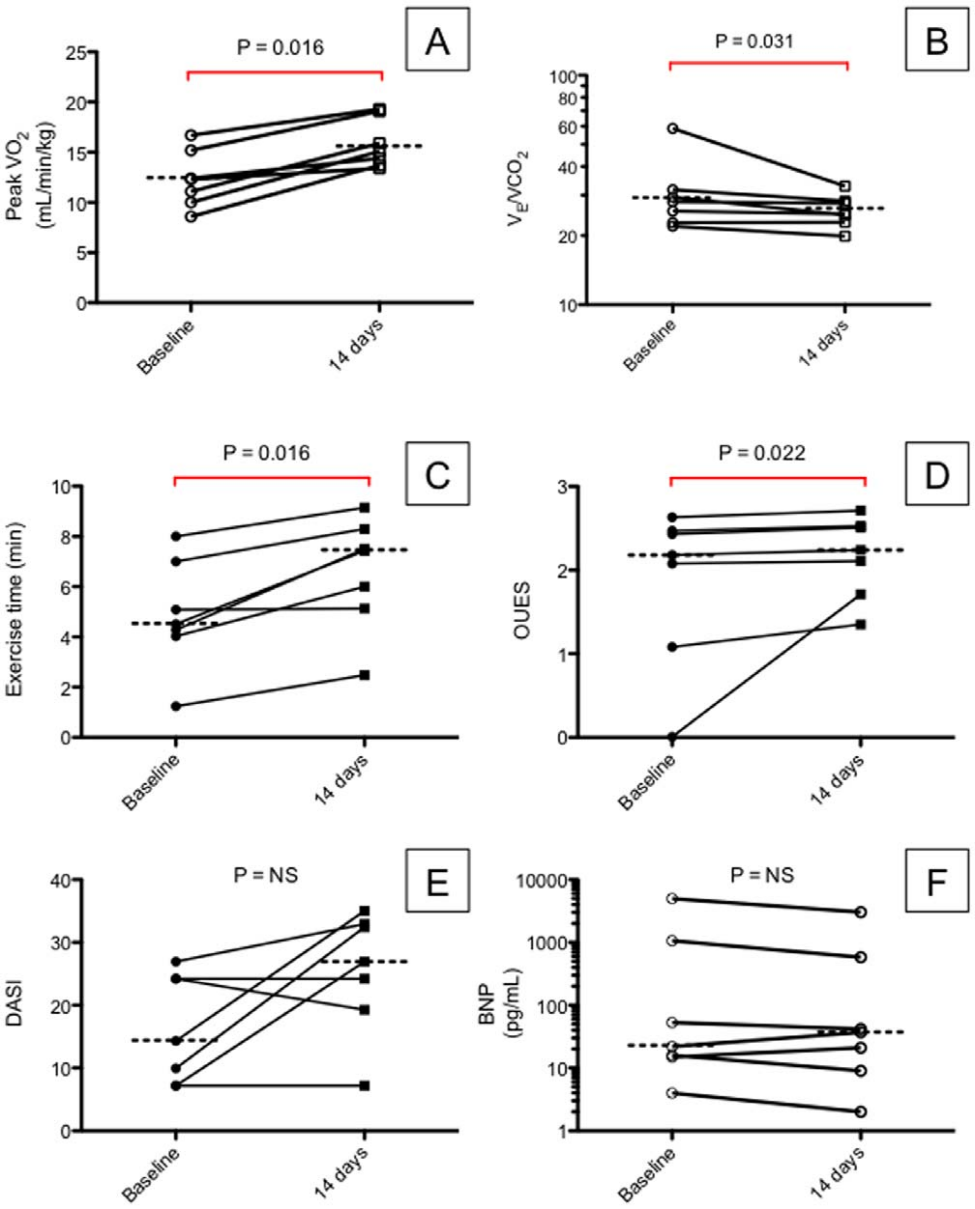
Cardiopulmonary exercise results. Two weeks treatment with anakinra improved multiple measures of cardipulmonary exercise performance. Individual panels show paired data for (A) peak oxygen consumption (peak VO2), (B) the slope of minute ventilation over carbon dioxide production (V_E_/VCO_2_), (C) exercise time, (D) oxygen uptake efficiency slope (OUES), (E) the Duke Activity Status Index (DASI), and (F) brain natriuretid peptide (BNP).

**Figure 6:**
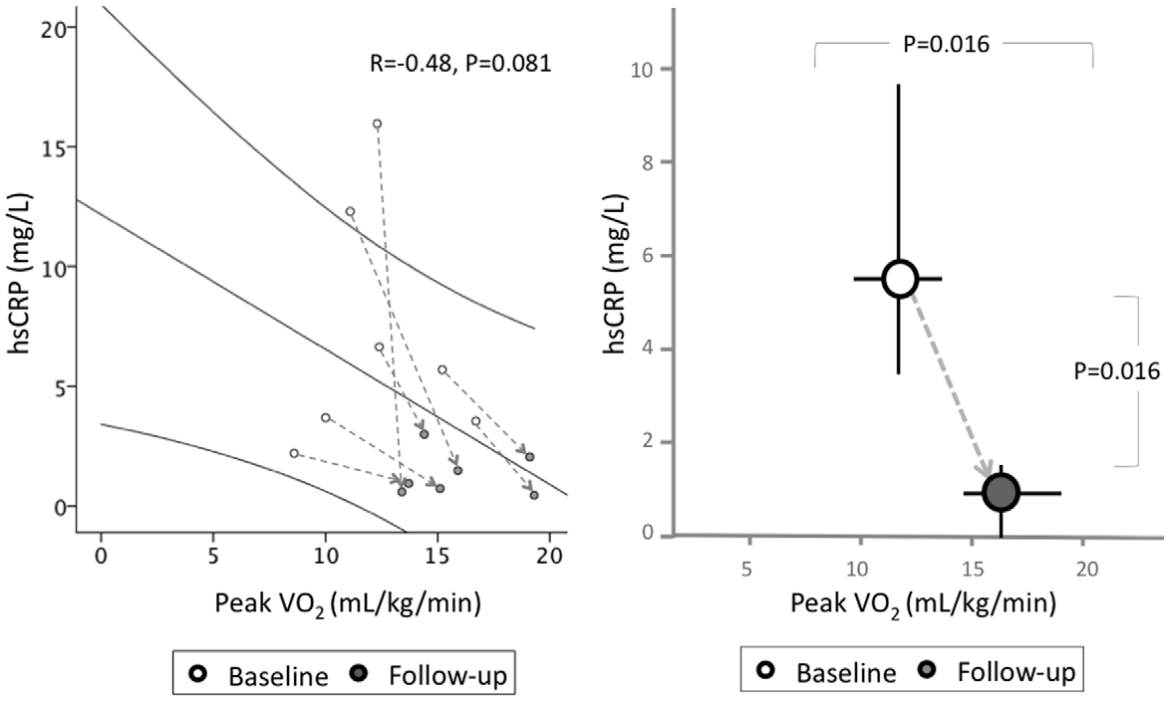
Correlation between peak VO2 and hsCRP. Two weeks treatment with anakinra produced independent benefits on both peak VO2 (increase) and hsCRP (decrease), however, the correlation between the two did not achieve statistical significance. White circles represent baseline values and dark circles represent follow-up values. The left panel also displays the linear Spearman’s correlation and 75% confidence interval. The right panel shows the change in median values for baseline and follow-up measurements.

All 7 patients experienced improvement in peak VO_2_ and 6 out of 7 patients experienced improvement in the VE/VCO_2_ slope following 2 weeks treatment with anakinra (Figure 5). All 7 patients experienced improvements in secondary endpoints of exercise time and oxygen utilization efficiency score. There were trends toward improvement in both DASI score (increased score) and BNP (reduced concentration), although these changes failed to reach statistical significance. The median peak VO_2_ improved from 12.3 [10.0, 15.2] to 15.1 [13.7, 19.3] mL·kg^-1^·min^-1^ (P = 0.016 vs. baseline), representing a relative improvement of 23%, 2.8 mL·kg^–1^·min^–1^, or nearly 1 metabolic equivalent (Table 3). The median V_E_/VCO_2_ slope improved from 28.1 [22.8, 31.7] to 24.9 [22.9, 28.3] (P = 0.031 vs. baseline), representing a relative improvement of 12% or 3.2 slope units. No significant changes occurred in resting heart rate (HR), maximum HR, resting blood pressure, maximum blood pressure, or heart rate recovery between baseline and 2 weeks CPX. Despite significant improvements in exercise parameters and significant reductions in hsCRP, the correlation between hsCRP and peak VO2 did not achieve statistical significance (Figure 6).

**Table 3:** Cardiopulmonary exercise parameters in pilot clinical study.

	Baseline (n = 7)	Final (n = 7)	P
Peak VO_2_	12.3 [10.6, 13.8]	15.1 [14.0, 17.5]	**0.016**
Slope V_E_VCO_2_	28.1 [24.2, 30.6]	24.9 [23.75, 28]	**0.031**
OUES	2.18 [1.58, 2.45]	2.24 [1.92, 2.52]	**0.022**
Exercise time (min)	4.51 [2.64, 4.8]	7.43 [5.6, 7.9]	**0.016**
RestHR (min^–1^)	75 [72, 80]	71 [68, 75]	0.11
MaxHR (min^–1^)	108 [83, 114]	102 [93, 126]	0.06
RestSBP (mmHg)	112 [102, 122]	113 [112, 120]	0.56
MaxSBP (mmHg)	125 [114, 155]	146 [123, 167]	0.09
RestDBP (mmHg)	76 [62, 80]	76 [70, 84]	0.36
MaxDBP (mmHg)	75 [65, 91]	84 [75, 92]	0.25

Abbreviations: DBP (diastolic blood pressure); OUES (oxygen utilization efficiency score); Peak VO_2_ (peak oxygen consumption); SBP (systolic blood pressure); V_E_/VCO_2_ (ventilatory efficiency).

### Effects of plasma from HF patients treated with anakinra for 2 weeks

**Figure 7:**
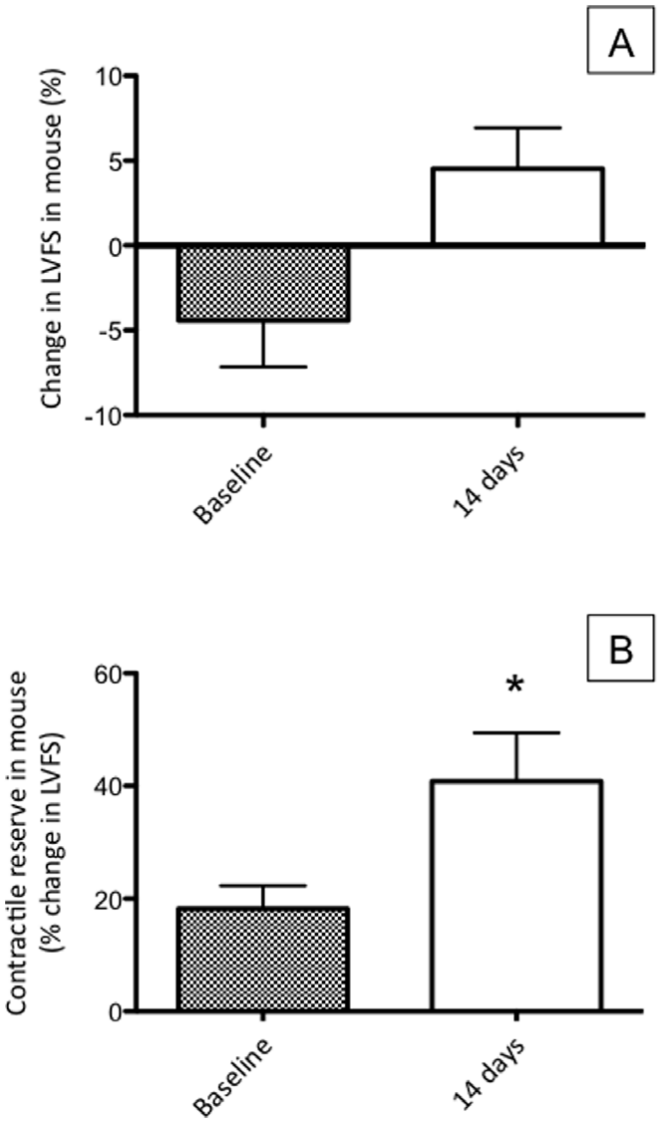
Effects of plasma from HF patients on LV function in healthy mice. Plasma from HF patients with high CRP was obtained before and after 2 weeks treatment with anakinra. After treatment with anakinra, plasma from HF patients with high CRP did not induce left ventricular dysfunction in mice as measured by (A) LV fractional shortening (LVFS) and (B) contractile reserve. Treatment with anakinra (in patients) neutralized the effects of HF plasma (in mice) similar to what was observed when pre-treatment with anakinra (injected into mice) neutralized the cardiac dysfunction observed in Figure 3. *P<0.05 versus Baseline.

Similar to the effects of pre-treating mice with anakinra, plasma from patients with HF–HCRP who were treated anakinra for 2 weeks no longer impaired contractile reserve when injected into healthy mice (Figure 7).

## Discussion

The role of inflammation in human HF has not been well established. Multiple pre-clinical investigations have demonstrated plausible mechanisms of pathologic inflammatory mediators, yet there are still no effective clinical treatments that target inflammation in HF.[5]–[12] Herein we describe that plasma from decompensated HF patients was sufficient to reproduce a phenotype of HF in healthy mice (impaired systolic function at rest and impaired contractile reserve) and that plasma from patients with stable HF and a high inflammatory burden was sufficient to reproduce a phenotype that was absent of symptoms at rest, but was still characterized by impaired contractile reserve. In both cases, the murine phenotypes mirrored the clinical phenotypes and were fully prevented with IL-1β blockade. Moreover, this same IL-1β blocking strategy translated to significant improvements in CPX performance over a 2-week period in a pilot study of HF patients.

Our results are consistent with previous pre-clinical studies in murine models of both ischemic and non-ischemic cardiomyopathy that demonstrate significant reductions in LV remodeling and improved cardiac function following IL-1 blockade. [8]–[12] Two small, randomized, double-blind trials have reported significant cardiovascular benefits following IL-1β blockade. Ikonomidis et al. reported improved vascular function (flow-mediated forearm vasodilation), coronary flow, and LV function (ratio of mitral annulus systolic/diastolic velocities) at 3 hours following randomization to a single anakinra injection versus placebo in patients with rheumatoid arthritis. [23] Within the same publication, investigators reported similar benefits in a separate population of rheumatoid arthritis patients following open-label assignment to 30 days treatment with anakinra versus prednisolone. In a recently completed study conducted in patients presenting with ST-elevation myocardial infarction, our group observed more favorable LV remodeling (smaller LV end systolic volume index) at 90 days following randomization to 14 days treatment with anakinra versus placebo. [24]

Compared to previous approaches to block inflammatory signaling in HF patients, IL-1β blockade represents a novel and finely targeted approach devoid of off-target effects. Prior attempts to inhibit inflammation in HF using corticosteroids or non-steroidal anti-inflammatory drugs (NSAIDs) have shown disappointing results. [25]–[27] Although both classes of drugs are powerful anti-inflammatory agents, both are substantially different from the IL-1β blockers and are inevitably non-specific in their effects as they simultaneously block multiple pathways, some of which may be protective. For example, both corticosteroids and NSAIDs promote fluid retention and hypertension that lead to worsening HF. Corticosteroids also induce hyperglycemia and activate the aldosterone receptor in the heart, which is directly linked to adverse remodeling and HF. Moreover, NSAIDs eliminate potential beneficial effects of locally produced prostaglandins and bradykinins. In contrast, IL-1β blockade is a selective mechanism that does not promote fluid retention, hypertension, hyperglycemia or any other significant metabolic alterations. No direct effects of IL-1β blockade on hemodynamic parameters, cardiac function, platelet function or coagulation have been reported in healthy volunteers. Therefore, IL-1β blockade may represent a safer approach.

As expected, IL-1β blockade in HF patients led to a significant reduction in hsCRP. The observation of potential reductions in IL-1β and IL-6 plasma concentrations without any observable change in TNFα concentration may be meaningful for two distinct reasons. First, the reduction of IL-1β plasma concentration in the presence of IL-1β blockade would be indicative of an underlying auto-inflammatory process in HF. In most physiological systems, administration of a receptor antagonist promotes agonist up-regulation through interruption of negative feedback loops. Conversely, in auto-inflammatory disorders, initial IL-1β activity primes the cellular apparatus for accelerated IL-1β production leading to a vicious cycle of inflammatory activity. [28]–[29] While HF has not, as of yet, been identified as an auto-inflammatory disorder, our observations would be consistent with this explanation. Second, the lack of TNFα effects may differentiate the effects of IL-1β blockade from the disappointments with TNFα blockers (i.e. etanercept and infliximab) in previous HF studies. [30] Although the 2 classes of anti-cytokine therapy are occasionally considered interchangeable for the treatment of rheumatic diseases, they block distinct signaling pathways that are mostly not convergent and have led to significant differences in efficacy, safety, and tolerability. Moreover, etanercept may exert partial agonist activity through a cytokine “reservoir” effect whereby cytokine binding proteins with prolonged elimination half-lives exhibit nontraditional dose–response curves that describe a maximum effective concentration. In addition, anti-TNF-α antibodies such as infliximab, may induce complement-mediated damage to cardiomyocytes expressing membrane-bound TNF-α.

There is an urgent need to identify novel treatment strategies for patients with HF. Despite significant advances in primary and secondary prevention strategies, HF remains a leading cause of hospitalization among US patients. [31] Poor aerobic exercise capacity and ventilatory inefficiency are common findings among HF patients and impose significant detriments to quality of life. [32] Moreover, quantifiable measures of exercise capacity such as peak VO_2_ and the V_E_/VCO_2_ slope represent strong independent predictors of HF mortality and hospitalization. [33] In fact, these aforementioned CPX variables have consistently proven to be amongst the strongest predictors of adverse events in this chronic disease population. [34] Given the prognostic implications of improvement in CPX variables, the results of the present study are particularly compelling. We further emphasize that these benefits occurred *in addition* to standard medical therapy including angiotensin converting enzyme inhibitors, β-AR blocker, diuretics, and aldosterone antagonists.

From a mechanistic standpoint, numerous studies have linked both a lower peak VO_2_ and higher V_E_/VCO_2_ slope to diminished cardiac function in patients with HF. [35] Therefore, the improvements in these CPX variables observed in the current investigation may be linked to IL-1β blockade-induced improvements in cardiac function. In this context, the degree of CPX improvements induced by IL-1β blockade treatment may be used to gauge therapeutic efficacy, if supported by future investigations.

There are numerous limitations to our current investigation. First, we were not able to elucidate the signaling mechanism of IL-1-induced systolic dysfunction. We observed a delayed, reversible LV systolic dysfunction coupled with a blunted response to isoproterenol. These findings may be explained by multiple potential mechanisms of β-adrenergic receptor dysregulation that have been published in cellular models of IL-1β signaling. [13]–[14] Second, we recognize that IL-1β is not the only circulating factor in human plasma with the potential to alter cardiac function. Instead of quantifying *all* of the numerous signaling molecules present in HF plasma, we focused our experiments on the physiologic effects of HF plasma that were susceptible to IL-1β blockade. While this approach does not reveal the comprehensive network of signaling molecules that may contribute to the phenotype, it does identify IL-1β as a critical mediator of the cardiac dysfunction. Third, our clinical study was not designed as a definitive evaluation of IL-1β blockade on cardiopulmonary exercise. Instead, we designed a pilot study to test the safety and feasibility of anakinra in a stable, ambulatory, HF population. The lack of randomization in the study limits the interpretation of study results to imply a definitive cause-and-effect relationship between anakinra treatment and changes in cardiopulmonary exercise capacity. We therefore report these findings as proof-of-concept for an *association* between anakinra treatment and the significant improvements observed in these patients. Fourth, HF patients are notoriously unstable and often progress through multiple periods of improvement or deterioration in physical symptoms. Any changes observed in non-randomized studies are therefore subject to increased scrutiny to demonstrate the “stability” of the HF population and the reliability of the baseline measurements. We therefore selected a population of ambulatory HF patients free from recent hospitalizations or medications changes and a relatively short study window to minimize the potential of rapid deteriorations or improvements in cardiopulmonary exercise performance over the course the study. We also relied on the use of objective, quantifiable measures of CPX performance (peak VO_2_ and the V_E_/VCO_2_ slope) that have been the most rigorously validated in previous studies of HF patients. [33] Further studies will soon be underway to confirm these findings and evaluate the persistence of CPX benefits with prolonged IL-1β blockade in a randomized, double-blind clinical trials accompanied by cardiac imaging to evaluate direct changes in cardiac dimension and function. Patients will receive treatment for up to 12 weeks with IL-1β blockade, followed by a prolonged follow-up (i.e. washout period). 

In summary we report the presence of a circulating factor in the plasma of human HF patients that is sufficient to induce cardiac dysfunction in healthy mice and susceptible to IL-1β blockade. Furthermore, we report the safety and feasibility of IL-1β blockade over the course of 2 weeks in stable HF patients with elevated hsCRP. Treatment with anakinra was associated with a significant reduction in hsCRP and significant improvements in aerobic capacity, ventilatory efficiency, and total exercise time. These findings suggest that IL-1β activity contributes to exercise intolerance in patients with systolic HF and identifies IL-1β blockade as a potential novel strategy for pharmacologic intervention.

## Supporting Information

Protocol S1 **Trial Protocol.**
(PDF)

Appendix S1 **The Duke Activity Status Index (DASI) questionnaire.**
(DOCX)

## References

[1] Jessup M, Abraham WT, Casey DE, Feldman AM, Francis GS (2009). 2009 focused update: ACCF/AHA Guidelines for the Diagnosis and Management of Heart Failure in Adults: a report of the American College of Cardiology Foundation/American Heart Association Task Force on Practice Guidelines: developed in collaboration with the International Society for Heart and Lung Transplantation.. Circulation.

[2] Roger VL, Go AS, Lloyd-Jones DM, Adams RJ, Berry JD (2011). Heart disease and stroke statistics–2011 update: a report from the American Heart Association.. Circulation.

[3] Deswal A, Petersen NJ, Feldman AM, Young JB, White BG (2001). Cytokines and cytokine receptors in advanced heart failure: an analysis of the cytokine database from the Vesnarinone trial (VEST).. Circulation.

[4] Araújo JP, Lourenço P, Azevedo A, Friões F, Rocha-Gonçalves F (2009). Prognostic value of high-sensitivity C-reactive protein in heart failure: a systematic review.. J Card Fail.

[5] Long CS (2001). The role of interleukin-1 in the failing heart.. Heart Fail Rev.

[6] Dinarello CA (2009). Immunological and inflammatory functions of the interleukin-1 family.. Annu Rev Immunol.

[7] Bujak M, Frangogiannis NG (2009). The role of IL-1 in the pathogenesis of heart disease.. Arch Immunol Ther Exp (Warsz).

[8] Abbate A, Salloum FN, Vecile E, Das A, Hoke NN (2008). Anakinra, a recombinant human interleukin-1 receptor antagonist, inhibits apoptosis in experimental acute myocardial infarction.. Circulation.

[9] Van Tassell BW, Varma A, Salloum FN, Das A, Seropian IM (2010). Interleukin-1 trap attenuates cardiac remodeling after experimental acute myocardial infarction in mice.. J Cardiovasc Pharmacol.

[10] Abbate A, Salloum FN, Van Tassell BW, Vecile E, Toldo S (2011). Alterations in the Interleukin-1/Interleukin-1 receptor antagonist balance modulate cardiac remodeling following acute myocardial infarction in the mouse.. PLoS ONE.

[11] Abbate A, Van Tassell BW, Seropian IM, Toldo S, Robati R (2010). Interleukin-1beta modulation using a genetically engineered antibody prevents adverse cardiac remodelling following acute myocardial infarction in the mouse.. Eur J Heart Fail.

[12] Bujak M, Dobaczewski M, Chatila K, Mendoza LH, Li N (2008). Interleukin-1 receptor type I signaling critically regulates infarct healing and cardiac remodeling.. Am J Pathol.

[13] Liu SJ, Zhou W, Kennedy RH (1999). Suppression of beta-adrenergic responsiveness of L-type Ca2+ current by IL-1beta in rat ventricular myocytes.. Am J Physiol.

[14] Gulick T, Chung MK, Pieper SJ, Lange LG, Schreiner GF (1989). Interleukin 1 and tumor necrosis factor inhibit cardiac myocyte β-adrenergic responsiveness.. Proc Natl Acad Sci U S A.

[15] Schiller NB, Shah PM, Crawford M, DeMaria A, Devereux R (1989). Recommendations for quantitation of the left ventricle by two-dimensional echocardiography. American Society of Echocardiography Committee on Standards, Subcommittee on Quantitation of Two-Dimensional Echocardiograms.. J Am Soc Echocardiogr.

[16] Syed F, Diwan A, Hahn HS (2005). Murine echocardiography: a practical approach for phenotyping genetically manipulated and surgically modeled mice.. J Am Soc Echocardiogr.

[17] Hlatky MA, Boineau RE, Higginbotham MB, Lee KL, Mark DB (1989). A brief self-administered questionnaire to determine functional capacity (the Duke Activity Status Index).. Am J Cardiol.

[18] Arena R, Humphrey R, Peberdy MA, Madigan M (2003). Predicting peak oxygen consumption during a conservative ramping protocol: implications for the heart failure population.. J Cardiopulm Rehabil.

[19] Baba R, Nagashima M, Goto M, Nagano Y, Yokota M (1996). Oxygen intake efficiency slope: a new index of cardiorespiratory functional reserve derived from the relationship between oxygen consumption and minute ventilation during incremental exercise.. Nagoya J Med Sci.

[20] Gibbons RJ, Balady GJ, Bricker JT, Chaitman BR, Fletcher GF (2002). ACC/AHA 2002 guideline update for exercise testing: summary article. A report of the American College of Cardiology/American Heart Association Task Force on Practice Guidelines (Committee to Update the 1997 Exercise Testing Guidelines).. J Am Coll Cardiol.

[21] Tei C, Ling LH, Hodge DO, Bailey KR, Oh JK (1995). New index of combined systolic and diastolic myocardial performance: a simple and reproducible measure of cardiac function–a study in normals and dilated cardiomyopathy.. J Cardiol.

[22] Broberg CS, Pantely GA, Barber BJ, Mack GK, Lee K (2003). Validation of the myocardial performance index by echocardiography in mice: a noninvasive measure of left ventricular function.. J Am Soc Echocardiogr.

[23] Ikonomidis I, Lekakis JP, Nikolaou M, Paraskevaidis I, Andreadou I (2008). Inhibition of interleukin-1 by anakinra improves vascular and left ventricular function in patients with rheumatoid arthritis.. Circulation.

[24] Abbate A, Kontos MC, Grizzard JD, Biondi-Zoccai GG, Van Tassell BW (2010). Interleukin-1 blockade with anakinra to prevent adverse cardiac remodeling after acute myocardial infarction (Virginia Commonwealth University Anakinra Remodeling Trial [VCU-ART] Pilot study).. Am J Cardiol.

[25] LeGal YM, Morrissey LL (1990). Methylprednisolone interventions in myocardial infarction: a controversial subject.. Can J Cardiol.

[26] Kones RJ (1975). Glucocorticoid therapy for acute myocardial infarction.. Acta Cardiol.

[27] Judgutt BI, Basualdo CA (1989). Myocardial infarct expansion during indomethacin or ibuprofen therapy for symptomatic post infarction pericarditis. Influence of other pharmacologic agents during early remodeling.. Can J Cardiol.

[28] Dinarello CA (2010). IL-1: discoveries, controversies and future directions.. Eur J Immunol.

[29] Mezzaroma E, Toldo S, Farkas D, Seropian IM, Van Tassell BW (2011). The inflammasome promotes adverse cardiac remodeling following acute myocardial infarction in the mouse.. *Proc Natl Acad Sci U S A*.

[30] Mann DL (2005). Targeted anticytokine therapy and the failing heart.. Am J Cardiol.

[31] Jencks SF, Williams MV, Coleman EA (2009). Rehospitalizations among patients in the Medicare fee-for-service program.. New Engl J Med.

[32] Task Force of the Italian Working Group on Cardiac Rehabilitation and Prevention (Gruppo Italiano di Cardiologia Riabilitativa e Prevenzione, GICR),; Working Group on Cardiac Rehabilitation and Exercise Physiology of the European Society of Cardiology (2006). Statement on cardiopulmonary exercise testing in chronic heart failure due to left ventricular dysfunction: recommendations for performance and interpretation Part III: Interpretation of cardiopulmonary exercise testing in chronic heart failure and future applications.. Eur J Cardiovasc Prev Rehabil.

[33] Balady GJ, Arena R, Sietsema K, Myers J, Coke L (2010). American Heart Association Exercise, Cardiac Rehabilitation, and Prevention Committee of the Council on Clinical Cardiology; Council on Epidemiology and Prevention; Council on Peripheral Vascular Disease; Interdisciplinary Council on Quality of Care and Outcomes Research. Clinician’s Guide to cardiopulmonary exercise testing in adults: a scientific statement from the American Heart Association.. Circulation.

[34] Guazzi M, Arena R (2009). The impact of pharmacotherapy on the cardiopulmonary exercise test response in patients with heart failure: a mini review.. Curr Vasc Pharmacol;.

[35] Arena R, Myers J, Guazzi M (2008). The clinical and research applications of aerobic capacity and ventilatory efficiency in heart failure: an evidence-based review.. Heart Fail Rev.

