# Supplementary material for: Enhanced Interleukin-1 Activity Contributes to Exercise Intolerance in Patients with Systolic Heart Failure
Source: PLoS One. 2012 Mar 16;7(3):e33438. doi: 10.1371/journal.pone.0033438 (PMC3306393; doi:10.1371/journal.pone.0033438)
Supplement: Protocol S1 — Trial Protocol. (PDF) [file pone.0033438.s001.pdf]

VCU RESEARCH PLAN TEMPLATE

Use of this template is required to provide your VCU Research Plan to the IRB. Your responses should be written in terms for the non-scientist to understand. If a detailed research protocol (e.g., sponsor's protocol) exists, you may reference that protocol. NOTE: **If that protocol does not address all of the issues outlined in each Section Heading, you must address the remaining issues in this Plan. It is NOT acceptable to reference a research funding proposal**.

ALL Sections of the Human Subjects Instructions must be completed with the exception of the Section entitled "Special Consent Provisions." Complete that Section if applicable. When other Sections are not applicable, list the Section Heading and indicate "N/A."

NOTE: **The Research Plan is required with ALL Expedited and Full review submissions and MUST follow the template, and include version number or date, and page numbers.**

DO NOT DELETE SECTION HEADINGS OR THE INSTRUCTIONS.

I. TITLE

Pilot Study of the Safety and Efficacy of Anakinra (recombinant human Interleukin-1 receptor antagonist) in Heart Failure

II. RESEARCH PERSONNEL

A. In the table below (add additional rows as needed), indicate: (1) all project personnel including the principal investigator and individuals from other institutions, (2) their qualifications, and (3) a brief description of their role or responsibilities on the study.**

**** Personnel list should include anyone engaged in the research (VCU & non-VCU personnel) including independent investigators. Engaged means interacting or intervening with research participants and/or having access to identifiable private information about participants. See OHRP's guidance on "Engagement of Institutions in Research" at http://www.hhs.gov/ohrp/humansubjects/guidance/engage08.html.**

NAME OF INDIVIDUAL	INSTITUTION	QUALIFICATIONS	RESPONSIBILITIES
Benjamin Van Tassell	Virginia Commonwealth University	Pharm.D.	Principal investigator. Responsible for overall design and conduct of study. Screen/consent potential research subjects. Conduct patient counseling regarding study medication. Data analysis and interpretation. Study coordinator (administrative responsibilities).
Antonio Abbate	Virginia Commonwealth University	MD, Ph.D.	Medically responsible investigator. Responsible for patient safety and clinical follow-up at all patient visits. Screen/consent potential research subjects. Conduct patient counseling regarding study medication. Assist with data analysis.
Ross Arena	Virginia Commonwealth University	Ph.D.	Cardiopulmonary exercise therapist. Responsible for exercise testing and assistance with data analysis

NOTE: If an independent investigator is "engaged," and the research involves a DIRECT FEDERAL award made to VCU (or

application for such), the independent investigator must sign a formal written agreement with VCU certifying terms for the protection of human subjects. For an agreement to be approved: (1) the PI must directly supervise all of the research activities, (2) agreement must follow the ORSP template, (3) IRB must agree to the involvement of the independent investigator, AND (4) agreement must be in effect prior to final IRB approval.

B. Describe the process that you will use to ensure that all persons assisting with the research are adequately informed about the protocol and their research-related duties and functions.

All individuals involved with the project know their assigned duties and the research protocol. All personnel assisting with the research will be briefed by the principal investigator on the research process during regular study group meetings. The investigators will maintain regular contact throughout the course of the study.

III. Conflict of Interest

Describe how the principal investigator and sub/co-investigators might benefit from the subject's participation in this project or completion of the project in general. Do not describe (1) academic recognition such as publications or (2) grant or contract based support of VCU salary commensurate with the professional effort required for the conduct of the project

No conflicts of interest.

IV. Resources

Briefly describe the resources committed to this project including: (1) time available to conduct and complete the research, (2) facilities where you will conduct the research, (3) availability of medical or psychological resources that participants might require as a consequence of the research (if applicable), and (4) financial support.

(1) The timeline to complete the research is 6 months. The investigators all have protected research time to devote to the study. The PI has been selected as a K12 Scholar by the VCU Center for Clinical and Translational Research that will guarantee 75% of his time/salary to conduct research and will provide start-up funds to cover the costs of the study. Although not scripted in the K12 Scholar application, we estimate that the PI's efforts toward this study will require approximately 20% of his total effort (4 hours/week for patient screening/enrollment [10%], plus 2-4 additional hours per week for all other duties), This 20% effort is well-within the 75% of time guaranteed in the K12 Scholar Award. Dr. Abbate also has 50% of his total effort protected for research activities by the Division of Cardiology. Dr. Abbate will assist with patient screening/enrollment as needed during the course of his regular Cardiology Fellows' Clinic (VCU Medical Center, Ambulatory Care Center, 4th floor, Monday afternoons). Cardiopulmonary testing will be performed at

(2) All patient screening will take place at the Cardiology Fellows' Clinic (VCU Medical Center, Ambulatory Care Center, 4th floor, Monday afternoons) for which Dr. Abbate is the attending physician. Aerobic exercise testing will be conducted at the Heart Station at VCU Medical Center.
(3) All patient contact will occur in standard clinical practice settings (Cardiology Fellows' Clinic, Heart Station, Cardiopulmonary Rehab Center). Standard medical resources (identical to those available to regular patients) will be available in case of unanticipated problems that might arise during research.
(4) The PI has received start-up funds ($25,000/year) from the VCU Center for Clinical and Translational Research. A portion of these start-up funds will be used to pay for the study (total estimated cost of the study is approximately $10,000). Financial resources available for the study may be verified with Sherry Bremer (sbremer@mcvh-vcu.edu), who is the fiscal administrator for the award program.

V. Hypothesis

Briefly state the problem, background, importance of the research, and goals of the proposed project.

Heart failure (HF) is a complex clinical syndrome characterized by dyspnea, fatigue, and declining cardiac function.[1] Poor exercise capacity is common finding among HF patients and imposes a significant detriment to quality of life.[1] Quantifiable measures of exercise capacity such as peak oxygen consumption (peak VO_2) and the minute ventilation and carbon dioxide production slope (V_E/VCO_2) represent strong independent predictors of HF mortality and hospitalization.[2-3]

Despite advances in HF diagnosis and treatment, the incidence of HF morbidity and mortality continue to rise, suggesting that the current treatment paradigm fails to interrupt one or more key pathologic mechanisms.[1] A significant correlation exists among HF patients between declining functional class and increasing levels of inflammatory cytokines.[4,5] While the inflammatory basis of HF remains poorly understood, Interleukin-1β (IL-1β) has recently been identified as a key modulator of cardiac dysfunction, atherosclerosis, ventricular remodeling, and cardiomyocyte apoptosis.[6,7]

IL-1 receptor antagonist is a naturally occurring protein in the body that blocks IL-1 activity. Anakinra is a recombinant form of IL-1 receptor antagonist that is FDA approved for the treatment of rheumatoid arthritis. Recently, the Investigators completed a pilot clinical trial in patients hospitalized with ST segment elevation acute myocardial infarction (STEMI) in which anakinra lead to improved cardiac remodeling at 3 months after discharge.[7] The current research is designed to investigate the safety and efficacy of anakinra (recombinant human IL-1 receptor antagonist) in heart failure patients. We hypothesize that blockade of IL-1 will improve exercise capacity in patients with symptomatic heart failure.

1. Jessup M, Abraham WT, Casey DE, Feldman AM, Francis GS, Ganiats TG, Konstam MA, Mancini DM, Rahko PS, Silver MA, Stevenson LW, Yancy CW. 2009 focused update: ACCF/AHA Guidelines for the Diagnosis and Management of Heart Failure in Adults: a report of the American College of Cardiology Foundation/American Heart Association Task Force on Practice Guidelines: developed in collaboration with the International Society for Heart and Lung Transplantation. *Circulation*. 2009;119:1977-2016.
2. Sarullo FM, Fazio G, Brusca I, Fasullo S, Paterna S, Licata P, Novo G, Novo S, Di Pasquale P. Cardiopulmonary Exercise Testing in Patients with Chronic Heart Failure: Prognostic Comparison from Peak VO2 and VE/VCO2 Slope. *Open Cardiovasc Med J*. 2010;4:127-34.
3. Arena R, Myers J, Aslam SS, Varughese EB, Peberdy MA. Peak VO2 and VE/VCO2 slope in patients with heart failure: a prognostic comparison. *Am Heart J*. 2004;147:354-60.
4. Deswal A, Petersen NJ, Feldman AM, Young JB, White BG, Mann DL. Cytokines and cytokine receptors in advanced heart failure: an analysis of the cytokine database from the Vesnarinone trial (VEST). *Circulation*. 2001;103:2055-9.
5. Long CS. The role of interleukin-1 in the failing heart. *Heart Fail Rev*. 2001;6(2):81-94.
6. Bujak M, Frangogiannis NG. The role of IL-1 in the pathogenesis of heart disease. *Arch Immunol Ther Exp (Warsz)*. 2009;57:165-76
7. Abbate A, Kontos MC, Grizzard JD, Biondi-Zoccai GG, Van Tassell BW, Robati R, Roach LM, Arena RA, Roberts CS, Varma A, Gelwix CC, Salloum FN, Hastillo A, Dinarello CA, Vetrovec GW; VCU-ART Investigators. Interleukin-1 blockade with anakinra to prevent adverse cardiac remodeling after acute myocardial infarction (Virginia Commonwealth University Anakinra Remodeling Trial [VCU-ART] Pilot study). *Am J Cardiol*. 2010;105:1371-1377

VI. Specific Aims

This will be non-randomized, single-arm pilot study to measure the effects of IL-1 blockade in patients with stable heart failure. The primary endpoint is the change in exercise capacity among stable heart failure patients (n = 10) following 14-days treatment with daily doses of anakinra 100 mg (SC, subcutaneous).

CO-PRIMARY ENDPOINTS

1) Median interval change from baseline in peak VO_2 at 14 days following daily doses of anakinra 100 mg SC

2) Median interval change from baseline in the minute ventilation and carbon dioxide production (VE/VCO2 slope) at 14 days following daily doses of anakinra 100 mg SC

SECONDARY ENDPOINTS

1) Interval change from baseline in biomarkers (high-sensitivity C-reactive protein, whole blood assay, brain natriuretic peptide) from baseline to 2 weeks

2) Interval change in HF symptoms as measured by Duke Activity Status Index (DASI) from baseline to 2 weeks

3) Correlation between interval changes in hsCRP, VO_2, and VE/VCO_2 at baseline and 2 weeks

4) Rate of adverse events and hospitalizations during 2-week duration of study

VII. Background and Significance

Include information regarding pre-clinical and early human studies. Attach appropriate citations.

IL-1β is the prototypal inflammatory cytokine involved in virtually every inflammatory response in the body. IL-1 acts as an acute phase reactant during tissue injury and becomes chronically elevated in HF.[8-10] In our own laboratory, we have recently described that IL-1β is sufficient to induce LV systolic dysfunction that closely parallels the cardiac dysfunction observed in HF.[11] Most notable among these findings was the observation that <u>IL-1β blunted the contractile response to isoproterenol (β adrenergic receptor agonist)</u>, a surrogate measure of exercise capacity.[12] We also found that among mice with ischemic heart failure (10-weeks post surgical coronary artery ligation), <u>a short course of IL-1β blockade was sufficient to improve contractile response to isoproterenol.</u>

Agents of IL-1 blockade have been FDA-approved for the treatment of inflammatory disorders (such as rheumatoid arthritis and cryopyrin-associated periodic syndromes) for many years. More recently, our group completed a pilot study to evaluate a recombinant IL-1 receptor antagonist (IL-1Ra, anakinra) in patients with acute myocardial infarction. In these patients, IL-1 blockade with anakinra showed a slight improvement in ventricular remodeling versus patients treated with placebo.[7] Antibodies targeted toward IL-1β have also been evaluated in patients with diabetes mellitus. In these phase I/II studies, IL-1 blockade showed a statistically significant reduction in hemoglobin A1c and no significant increase in adverse effects.[13]

In a double-blind, randomized, placebo-controlled, anakinra improved vascular function when used in patients with rheumatoid arthritis.[14] When compared to placebo, the investigators found improvements in cardiac and endothelial function at **<u>3 hours after a single subcutaneous injection of anakinra</u>**. In the same publication, investigators reported a separate randomized study of rheumatoid arthritis patients who experienced similar improvements in cardiac and endothelial function upon completion of 30 days treatment with anakinra versus prednisolone.[14]

Our group recently conducted a preliminary analysis of patients hospitalized with heart failure to evaluate the presence of C-reactive protein (CRP) levels, a surrogate for IL-1β activity (Unpublished data, VCU IRB

HM12494, PI Abbate). To date, all enrolled subjects have exhibited elevated CRP levels throughout their hospital stay, suggesting that patients with acute decompensated heart failure have a sustained acute inflammatory response unrelated to biochemical and echocardiographic parameters and not responsive to conventional treatments (including medications such as diuretics, angiotensin-converting enzyme inhibitors, angiotensin receptor blockers, nesiritide, or beta-adrenergic blockers). This inflammatory response may represent a modifiable factor in determining the clinical course of heart failure hospitalizations.

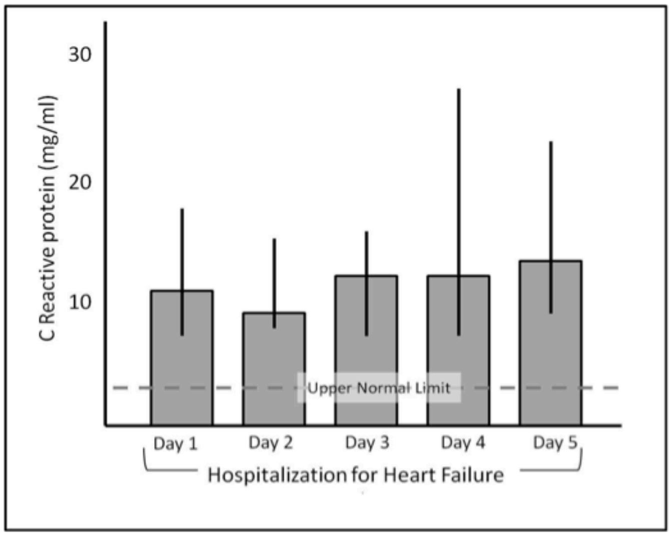

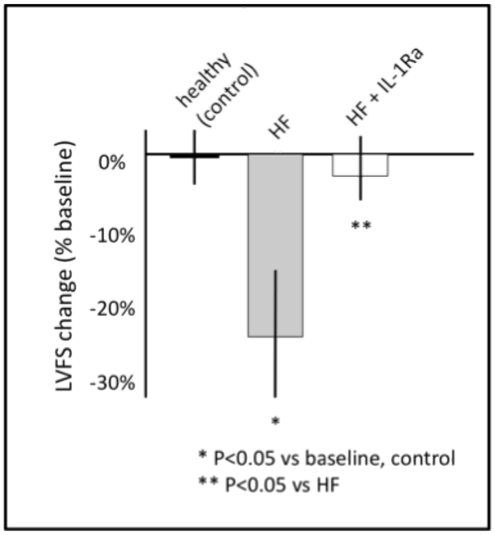

Figure 1 (left). Patients with ADHF exhibited CRP levels above upper normal limit throughout their hospital stay.
Figure 2 (right). Blood from patients with ADHF (or healthy control patients) was injected into healthy mice. Mice injected with blood from ADHF patients experienced significant cardiac dysfunction. Pre-treatment with anakinra (IL-1ra) prevented the cardiac dysfunction.

Plasma from these same HF patients (or plasma from healthy volunteers) was then injected into healthy mice followed by echocardiographic measurement of cardiac function (Unpublished data, VCU IUCAC AD20114, PI Van Tassell). At 4 hours after injection, LV fractional shortening remained unchanged in mice that received plasma from healthy volunteers (0±4%), but was significantly reduced in mice that received that plasma from HF patients (-25±6%, P=0.026 versus control). This cardiac dysfunction was prevented, however, by pre-treatment with an IL-1β blocking agent (anakinra [IL-1Ra] 10 mg/kg given 30 minutes prior to plasma injection). <u>These findings suggest the presence of a circulating factor in HF patients that (1) is sufficient to induce cardiac dysfunction and (2) is dependent on IL-1 signaling</u>.

In summary, there are compelling data to suggest that (1) heart failure is characterized by poor cardiac function, blunted response to adrenergic stimulation, and poor exercise capacity; (2) IL-1β signaling is elevated in the plasma of patients with heart failure; (3) IL-1β is sufficient to induce cardiac dysfunction and blunted response to β-adrenergic receptor agonists in mice; (4) IL-1 blockade prevents cardiac dysfunction that results from murine injection with plasma from heart failure patients. We therefore propose that IL-1 blockade will improve exercise capacity in patients with symptomatic heart failure.

7. Abbate A, Kontos MC, Grizzard JD, Biondi-Zoccai GG, Van Tassell BW, Robati R, Roach LM, Arena RA, Roberts CS, Varma A, Gelwix CC, Salloum FN, Hastillo A, Dinarello CA, Vetrovec GW; VCU-ART Investigators. Interleukin-1 blockade with anakinra to prevent adverse cardiac remodeling after acute myocardial infarction (Virginia Commonwealth University Anakinra Remodeling Trial [VCU-ART] Pilot study). *Am J Cardiol*. 2010;105:1371-1377
8. Dinarello CA. Immunological and inflammatory functions of the interleukin-1 family. *Annu Rev Immunol.* 2009;27:519-50.
9. Bujak M, Frangogiannis NG. The role of IL-1 in the pathogenesis of heart disease. *Arch Immunol Ther Exp (Warsz)*. 2009;57(3):165-76. Epub 2009.
10. Patti G, D'Ambrosio A, Mega S, Giorgi G, Zardi EM, Zardi DM, Dicuonzo G, Dobrina A, Di Sciascio G. Early interleukin-1 receptor antagonist elevation in patients with acute myocardial infarction. *J Am Coll Cardiol*.

2004;43:35-8.

11. Van Tassell BW, Seropian IM, Harrington JL, Menna AC, Scharf AW, Whitaker S, Varma A, Salloum FN, Abbate A. Interleukin-1 induces myocardial systolic dysfunction in the mouse through a PI3Kγ dependent pathway. *Eur Heart J*. 2009;Suppl 1:3-1038.
12. Van Tassell BW, Toldo S, Mezzaroma E, Goyal N, Seropian IM, Abbate A. Interleukin-1β modulates cardiac contractility through desensitization of the β1-adrenergic receptor. [ABSTRACT – International Cytokine Society. 2010, Chicago, IL]
13. Owyang AM, Gross L, Shu L, Esposito L, Maedler K, Kantak S. XOMA 052, an anti-IL-1β antibody, preserves beta-cell function and reduces hyperglycemia in the diet-induced obesity mouse model of type 2 diabetes. [ABSTRACT – American Diabetes Asssociation. 2009, San Francisco, CA]
14. Ikonomidis I, Lekakis JP, Nikolaou M, Paraskevaidis I, Andreadou I, Kaplanoglou T, Katsimbri P, Skarantavos G, Soucacos PN, Kremastinos DT. Inhibition of interleukin-1 by anakinra improves vascular and left ventricular function in patients with rheumatoid arthritis. Circulation. 2008;117(20):2662-9.

VIII. Preliminary Progress/Data Report

If available.

N/A

IX. Research Method and Design

Include a brief description of the project design including the setting in which the research will be conducted and procedures. If applicable, include a description of procedures being performed already for diagnostic or treatment purposes.

Patients will be screened by the investigators in the Cardiology Fellows' Clinic at VCU Medical Center (Ambulatory Care Center, 4th floor) during the course of their regular clinical care. Those who meet entry criteria will be approached by Dr. Van Tassell and/or Dr. Abbate for enrollment. Potential subjects will be provided with the IRB-approved Informed Consent Form and given the opportunity to discuss the study with investigators. Enrolled subjects will then undergo complete screening for entry criteria (listed below), a blood draw, pregnancy testing (in women), and a brief physical exam. Where possible, investigators will rely on clinically available laboratory results; the remaining laboratory analyses will be ordered and billed to the study account.

Upon completion of screening/enrollment, subjects will be scheduled for Visit 1 at the Heart Station (or Cardiopulmonary Rehab Center) at VCU Medical Center. Prior to exercise, subjects will complete the Duke Activity Status Index (DASI) questionnaire. The DASI is a twelve question, yes/no, instrument that allows for the calculation of perceived functional capacity. Each question describes a different physical activity and asks the subjects if they feel they can perform the task. The questions are weighted according to their degree of physical exertion. The weighted values from the "yes" responses are summed to produce a score in metabolic equivalents.

A physician-supervised maximal aerobic exercise test will be administered by standard clinical personnel using a metabolic cart that is interfaced with a treadmill. All cardiopulmonary testing at the Heart Center or Cardiopulmonary Rehab Center is performed under the supervision of an attending cardiologist. A conservative ramping treadmill protocol will be used. Prior to each test, the oxygen and carbon dioxide sensors will be calibrated using gases of known oxygen, nitrogen, and carbon dioxide concentrations and the flow sensor will

American Heart Association guidelines support maximal aerobic testing in stable patients two weeks post myocardial infarction and thereafter.

Upon completion of exercise testing, subjects will return to the Cardiology Fellows' Clinic at VCU Medical Center (Visit 2) to review the results of the cardiopulmonary exercise test with Dr. Abbate. Subjects with ECG changes suggestive of coronary ischemia (or other excluded findings [see Exclusion Criteria]) will be excluded from participation and the subjects will be referred for appropriate medical care by Dr. Abbate. Remaining subjects will receive a 14-day supply of study medication (anakinra 100 mg SC daily for 14 days) and a printed copy of the FDA approved "Patient Information" packet for anakinra. This "Patient Information" packet describes detailed patient safety information as well as storage, disposal, and step-by-step administration instructions for anakinra injection. The investigators (Dr. Abbate and/or Dr. Van Tassell) will review this packet with the subjects to ensure comprehension prior to their departure from clinic. (Along with study medications, patients will also receive portable syringe disposal containers [about the size of 1-gallon milk jug] which the investigators will collect at the termination of the study for proper disposal.)

Subjects will return to the Heart Center (or Cardiopulmonary Rehab Center) at VCU Medical Center for Visit 3 upon completion of 14-days treatment with anakinra. At this visit, subjects will undergo a brief physical exam, blood draw, questionnaire (DASI), and repeat exercise testing. This will conclude subject participation in the study. Upon completion of the study, subjects will return to their normally scheduled care at the Cardiology Fellows' Clinic at VCU Medical Center. Subjects will be provided with complete copies of all study results upon request at their next scheduled visit at the Cardiology Fellows' Clinic.

X. PLAN FOR CONTROL OF INVESTIGATIONAL DRUGS, BIOLOGICS, AND DEVICES.

Investigational drugs and biologics: IF Investigational Drug Pharmacy Service (IDS) is not being used, attach the IDS confirmation of receipt of the management plan.

Investigational and humanitarian use devices (HUDs): Describe your plans for the control of investigational devices and HUDs including:
(1) how you will maintain records of the product's delivery to the trial site, the inventory at the site, the use by each subject, and the return to the sponsor or alternative disposition of unused product(s);
(2) plan for storing the investigational product(s)/ HUD as specified by the sponsor (if any) and in accordance with applicable regulatory requirements;
(3) plan for ensuring that the investigational product(s)/HUDs are used only in accordance with the approved protocol; and
(4) how you will ensure that each subject understands the correct use of the investigational product(s)/HUDs (if applicable) and check that each subject is following the instructions properly (on an ongoing basis).

All drugs will be stored in and dispensed by the VCU Investigational Drug Pharmacy.

We have received a letter from FDA confirming that no IND is required for this study (copy included with application packet).

XI. DATA ANALYSIS PLAN

For investigator–initiated studies.

Given an expected average peak VO_2 of 15 ± 3 mL/kg/min for patients with symptomatic HF, 10 subjects per group would provide 80% power to detect an improvement of 3.5 mL/kg/min (1 metabolic equivalent). An improvement of 1 metabolic equivalent is typically associated with a 13 – 15% reduction in mortality. Data will be presented as median and interquartile range and non-parametric tests will be used due to the potential

paired data with (alpha = 0.05).

Spearman's test will be used to define the association between interval changes in peak VO_2, VE/VCO_2 and DASI, hsCRP, and other potential secondary endpoints.

XII. Data and Safety Monitoring

- **If the research involves greater than minimal risk and there is no provision made for data and safety monitoring by any sponsor, include a data and safety-monitoring plan that is suitable for the level of risk to be faced by subjects and the nature of the research involved.**
- **If the research involves greater than minimal risk, and there is a provision made for data and safety monitoring by any sponsor, describe the sponsor's plan.**
- **If you are serving as a Sponsor-Investigator, identify the Contract Research Organization (CRO) that you will be using and describe the provisions made for data and safety monitoring by the CRO. Guidance on additional requirements for Sponsor-Investigators is available at http://www.research.vcu.edu/irb/wpp/flash/X-2.htm**

Given that this is a pilot study involving a limited number of patients (n=10), we have not convened a Data and Safety Monitoring Board (DSMB) for this study. Instead, our "Data and Safety Monitoring Plan" stipulates that all adverse events will be reported directly to the medically responsible investigator (Dr. Abbate), who will then determine appropriate medical treatment and/or referral. Any unanticipated problems will be promptly reported to the VCU IRB.

XIII. Multi-center Studies

If VCU is the lead site in a multi-center project or the VCU PI is the lead investigator in a multi-center project, describe the plan for management of information that may be relevant to the protection of subjects, such as reporting of unexpected problems, project modifications, and interim results.

N/A

XIV. Involvement of Non-VCU Institutions/Sites (Domestic and Foreign)

1. **Provide the following information for each non-VCU institution/site (domestic and foreign) that has agreed to participate:**
    - **Name of institution/site**
    - **Contact information for institution/site**
    - **Engaged in Research or not (if YES AND the research involves a DIRECT FEDERAL AWARD made to VCU, include FWA #). See OHRP's guidance on "Engagement of Institutions in Research" at http://www.hhs.gov/ohrp/humansubjects/guidance/engage08.html.** See VCU WPPs **http://www.research.vcu.edu/irb/wpp/flash/XVII-6.htm** and **http://www.research.vcu.edu/irb/wpp/flash/XVII-11.htm**.

Name of Institution	Contact Information for Site	Engaged (Y/N) and FWA # if applicable
N/A		

2. **Provide a description of each institution's role (whether engaged or not) in the research, adequacy of the facility (in order to ensure participant safety in the case of an unanticipated emergency), responsibilities of its agents/employees, and oversight that you will be providing in order to ensure adequate and ongoing protection of the human subjects. You should only identify institutions that have agreed to participate. If additional institutions agree to participate at a later time, they must be added by amendment to the protocol.**

N/A

XVI. Human Subjects Instructions

<u>ALL</u> sections of the Human Subjects Instructions must be completed with the exception of the section entitled

"Special Consent Provisions." Complete that section if applicable.

A. DESCRIPTION

Provide a detailed description of the proposed involvement of human subjects or their private identifiable data.

Patients will be screened by the investigators in the Cardiology Fellows' Clinic at VCU Medical Center (Ambulatory Care Center, 4th floor) during the course of their regular clinical care. Those who meet entry criteria

Fellows' Clinic at VCU Medical Center. Subjects will be provided with complete copies of all study results upon request at their next scheduled visit at the Cardiology Fellows' Clinic.

B. Subject Population

Describe the subject population in terms of sex, race, ethnicity, age, etc., and your access to the population that will allow recruitment of the necessary number of participants. Identify the criteria for inclusion or exclusion of all targeted populations and include a justification for any exclusions. Explain the rationale for the involvement of special cases of subjects, such as children, pregnant women, human fetuses, neonates, prisoners or others who are likely to be vulnerable. If you plan to allow for the enrollment of Wards of the State (or any other agency, institution, or entity), you must specifically request their inclusion and follow guidance in VCU IRB WPP XV-3: Wards and Emancipated Minors available at http://www.research.vcu.edu/irb/wpp/flash/XV-3.htm.

All patients over the age of 18 with (1) a confirmed diagnosis of heart failure, (2) a recent echocardiogram documenting LV ejection fraction <40%, and (3) hsCRP >2 mg/L.

EXCLUSION CRITERIA:
- Recent changes (previous 3 months) in HF maintenance medications (beta-blockers, angiotensin converting enzyme [ACE] inhibitors, aldosterone antagonists, vasodilators, cardiac glycosides, diuretics)
- Hospitalization for worsening HF or acute decompensated HF within the previous 12 months
- Anticipated need for cardiac resynchronization therapy (CRT) or automated-implantable cardioverter defibrillator (AICD)
- Angina or electrocardiograph (ECG) changes that limit maximum exertion during cardiopulmonary exercise testing or baseline ECG changes that limit the ability to detect ischemia (i.e. left bundle-branch block).
- Recent (<14 days) use of anti-inflammatory drugs (not including NSAIDs), chronic inflammatory disorder (including but not limited to rheumatoid arthritis, systemic lupus erythematosus), malignancy, active infection, or any comorbidity limiting survival or ability to complete the study
- Severe kidney dysfunction (eGFR <30 mL/min)
- Coagulopathy (INR >1.5), thrombocytopenia (<50,000/mm3), or leukopenia (absolute neutrophil count <1,500/mm3)
- Pregnancy (female patients will be required to take a urine pregnancy test)
- Latex or rubber allergy
- Inability to give informed consent

C. Research Material

Identify the sources of research material obtained from individually identifiable living human subjects in the form of specimens, records, or data. Indicate whether the material or data will be obtained specifically for research purposes or whether use will be made of existing specimens, records, or data.

Data will be collected from baseline medical history/exam, laboratory assessments, cardiopulmonary exercise testing and survey of heart failure symptoms (questionnaire) as described in the Research Methods and Design (above Section IX). Where possible, we will rely on existing clinical data.

D. Recruitment Plan

Describe <u>in detail</u> your plans for the recruitment of subjects including:
(1) how potential subjects will be identified (e.g., school personnel, health care professionals, etc),
(2) how you will get the names and contact information for potential subjects, and
(3) who will make initial contact with these individuals (if relevant) and how that contact will be done.
If you plan to involve special cases of subjects, such as children, pregnant women, human fetuses, neonates, prisoners or others who are likely to be vulnerable, describe any special recruitment procedures for these populations.

Patients will be screened by the investigators in the Cardiology Fellows' Clinic at VCU Medical Center (Ambulatory Care Center, 4th floor) during the course of their regular clinical care. Those who meet entry criteria will be approached by Dr. Van Tassell and/or Dr. Abbate for enrollment. Potential subjects will be provided with the IRB-approved Informed Consent Form and given the opportunity to discuss the study with investigators.

Enrolled subjects will then undergo complete screening for entry criteria (listed below), a blood draw, pregnancy test (if female), and brief physical exam. Where possible, investigators will rely on clinically available laboratory results; the remaining laboratory analyses will be ordered and billed to the study account.

E. Privacy of Participants

NOTE: Privacy refers to individuals and their interests in controlling access to their identities, their physical person, and how and what kind of information is obtained about them. Privacy also encompasses the interests of defined communities (e.g. those with a certain diagnosis or social circumstance) in controlling access to the group identity and information about the group or individuals as part of the group.

Describe how the privacy interests of subjects (and communities, if appropriate) will be protected including:
(1) in the research setting (e.g., in the identification, recruitment, and intervention settings) and
(2) with the information being sought and the way it is sought. For example, providing drapes or barriers, interviewing in a private room, and collecting only the amount of sensitive information needed for identification, recruitment, or the conduct of the study.

All study procedures will be carried out in two possible locations:

Clinic visits will be conducted in individual ("private") patient rooms at the Cardiology Fellows' Clinic at VCU Medical Center (Ambulatory Care Center, 4th floor). These rooms are accessible only to the clinical staff and are considered sufficiently private to conduct full physical exams and patient interviews during regular clinical practice.

Exercise testing will take place in the Heart Station (or Cardiopulmonary Rehab Center) at VCU Medical Center, which is also a standard patient-care setting. Tests will be conducted in individual patient rooms that are accessible only to the clinical staff and not accessible to the public.

F. Confidentiality of Data

NOTE: Confidentiality refers to the way private, identifiable information about a subject or defined community is maintained and shared.

Check all of the following precautions that will be used to maintain the confidentiality of identifiable information:

☒ Paper-based records will be kept in secure location and only accessed by authorized study personnel
☒ Electronic records will be made available only to those personnel in the study through the use of access controls and encryption
☒ Identifiers will be removed from study-related data (data is coded with a key stored in a separate secure location)
❑ For research involving web-based surveys, data is secured via passwords and encryption
❑ Audio or video recordings of subjects will be transcribed and then destroyed to prevent audio or visual identification. Note the date of destruction (e.g., 3 months from close of study; after transcription is determined to be error free).
❑ Obtaining a Certificate of Confidentiality
❑ Other precautions:

G. Potential Risks

Describe potential risks (physical, psychological, social, legal, or other) and assess their likelihood and seriousness. Where appropriate, describe alternative treatments and procedures that might be advantageous to the subjects.

Anakinra is generally well-tolerated with few side effects. The most likely side effects associated with the use of anakinra include:
- Headache (>10%)
- Injection site reaction (>10%) such as pain, irritation, redness at the site of injection
- Diarrhea (1-10%)
- Nausea (1-10%)

- Neutropenia (rare)

Despite potential neutropenia, anakinra use is not associated with increased risk of infection (meta-analyses suggest that anakinra may improve survival in patients with sepsis).

Other risks involved with the study include pain/infection from the blood draws, allergy to anakinra, and fatigue/discomfort from the cardiopulmonary exercise tests. These activities will be conducted under standard medical supervision to minimize the potential risks to subjects.

H. Risk Reduction

Describe procedures for protecting against or minimizing potential risk. Where appropriate, discuss provisions for ensuring necessary medical or professional intervention in the event of adverse events to the subjects. Describe the provisions for monitoring the data collected to ensure the safety of subjects, if any.

Anakinra has been FDA approved for treatment of rheumatoid arthritis for almost 10 years. Anakinra is generally well-tolerated with few side effects. To minimize potential risks, the study will be conducted over the minimum time course that is hypothesized to achieve objective improvement in cardiopulmonary exercise parameters (2 weeks). The time-course of this hypothesis is based on (1) a previous publication suggesting improvements in endothelial and cardiac function after a single dose of anakinra in rheumatoid arthritis patients[14]; (2) improved ventricular remodeling following 2-weeks treatment in a pilot study of patients with acute myocardial infarction[7]; and (3) animal studies, suggesting improvement in beta-agonist (isoproterenol) following 3-days treatment with anakinra (unpublished data, see section VII above).

All research procedures are being conducted in standard clinical practice settings. The cardiopulmonary exercise will be performed by trained personnel under physician supervision at the Heart Station (or Cardiopulmonary Rehab Center) at VCU Medical Center.

I. Additional Safeguards for vulnerable participants

Describe any additional safeguards to protect the rights and welfare of participants if you plan to involve special cases of subjects such as children, pregnant women, human fetuses, neonates, prisoners or others who are likely to be vulnerable.

Safeguards to protect the rights and welfare of participants might relate to Inclusion/Exclusion Criteria: ("Adults with moderate to severe cognitive impairment will be excluded." "Children must have diabetes. No normal controls who are children will be used.") Consent: ("Participants must have an adult care giver who agrees to the participant taking part in the research and will make sure the participant complies with research procedures." "Adults must be able to assent. Any dissent by the participant will end the research procedures.") Benefit: ("Individuals who have not shown benefit to this type of drug in the past will be excluded.").

N/A

J. Risk/Benefit

Discuss why the risks to participants are reasonable in relation to the anticipated benefits to subjects and in relation to the importance of the knowledge that may reasonably be expected to result. If a test article (investigational new drug, device, or biologic) is involved, name the test article and supply the FDA approval letter.

Anakinra is approved by the FDA for the indication of rheumatoid arthritis. Anakinra has shown dramatic benefits in multiple models of inflammatory cardiovascular disease and has shown a trend towards improved cardiovascular function in multiple clinical trials (rheumatoid arthritis, ST elevation myocardial infarction, diabetes). This study will use the exact same treatment regimen (dose, route, duration) that was used in the recently completed pilot study of patients with ST elevation myocardial infarction (Abbate et al. Am J Cardiol, 2010).

K. Compensation Plan

Compensation for participants (if applicable) should be described, including possible total compensation, pro-rating,

any proposed bonus, and any proposed reductions or penalties for not completing the project.

The patients will be paid $50 per visit. If any patients withdraw from the study before completion, they will be paid for the amount of time they were enrolled ($50 per visit). Patients who are consented, but do not meet complete entry criteria (i.e. hsCRP) will receive credit for one study visit ($50).

L. CONSENT ISSUES

1. CONSENT PROCESS

Indicate who will be asked to provide consent/assent, who will obtain consent/assent, what language (e.g., English, Spanish) will be used by those obtaining consent/assent, where and when will consent/assent be obtained, what steps will be taken to minimize the possibility of coercion or undue influence, and how much time will subjects be afforded to make a decision to participate.

Patients will be screened from the Cardiology Fellows' Clinic at VCU Medical Center (Ambulatory Care Center, 4th floor) during the course of their regular clinical care by the investigators listed on this form. Potential subjects will be given as much time as needed to review the consent form, ask questions, and consider their participation. All eligible patients will be asked for consent to participate in our study using our research consent form approved by the VCU IRB. We ask that patients take sufficient time to thoroughly read the consent form and ask any questions on any concerns they may have about the study or any language they do not understand.

2. SPECIAL CONSENT PROVISIONS

If some or all subjects will be cognitively impaired, or have language/hearing difficulties, describe how capacity for consent will be determined. Consider using the VCU Informed Consent Evaluation Instrument available at http://www.research.vcu.edu/irb/guidance.htm. If you anticipate the need to obtain informed consent from legally authorized representatives (LARs), please describe how you will identify an appropriate representative and ensure that their consent is obtained. Guidance on LAR is available at http://www.research.vcu.edu/irb/wpp/flash/XI-3.htm.

N/A

3. ASSENT PROCESS

If applicable, explain the Assent Process for children or decisionally impaired subjects. Describe the procedures, if any, for re-consenting children upon attainment of adulthood. Describe procedures, if any, for consenting subjects who are no longer decisionally impaired. Guidance is available at http://www.research.vcu.edu/irb/wpp/flash/XV-2.htm and http://www.research.vcu.edu/irb/wpp/flash/XVII-7.htm.

N/A

4. REQUESTS FOR WAIVERS OF CONSENT (COMPLETE IF REQUESTING ANY TYPE OF WAIVER OF CONSENT OR ASSENT)

4-A. REQUEST TO WAIVE SOME OR ALL ELEMENTS OF INFORMED CONSENT FROM SUBJECTS OR PERMISSION FROM PARENTS: A waiver of informed consent means that the IRB is not requiring the investigator to obtain informed consent OR the IRB approves a consent form that does not include or alters some/all of the required elements of consent. **Guidance is available at http://www.research.vcu.edu/irb/wpp/flash/XI-1.htm. NOTE: Waiver is not allowed for FDA-regulated research unless it meets FDA requirements for Waiver of Consent for Emergency Research (see below).**

4-A.1. Explain why a waiver or alteration of informed consent is being requested.

4-A.2. Describe how this study meets ALL FOUR of the following conditions for a waiver or alteration:

- The research involves no more than minimal risk to the participants. → Explain how your study meets this criteria:

- The waiver or alteration will not adversely affect the rights and welfare of participants. → Explain how your

study meets this criteria:

- The research could not practicably be carried out without the waiver or alteration. → Explain how your study meets this criteria:

- Will participants be provided with additional pertinent information after participation?
  ☐ Yes
  ☐ No → Explain why not:

4-B. REQUEST TO WAIVE DOCUMENTATION OF CONSENT: A waiver of documentation occurs when the consent process occurs but participants are not required to sign the consent form. Guidance is available at http://www.research.vcu.edu/irb/wpp/flash/wpp_guide.htm#XI-2.htm. One of the following two conditions must be met to allow for consenting without signed documentation. <u>Choose which condition is applicable and explain why (explanation required)</u>:

☐ The only record linking the participant and the research would be the informed consent form. The principal risk to the participant is the potential harm resulting from a breach of confidentiality. Each participant will be asked whether he/she wants documentation linking the participant with the research and the participants wishes will govern. → Explain how your study fits into the category:

☐ The research presents no more than minimal risk of harm to participants & involves no procedures for which signed consent is normally required outside of the research context. → Explain how your study fits into the category:

4-C. REQUEST TO WAIVE SOME OR ALL ELEMENTS OF ASSENT <u>FROM CHILDREN ≥ AGE 7 OR FROM DECISIONALLY IMPAIRED INDIVIDUALS:</u> A waiver of assent means that the IRB is not requiring the investigator to obtain assent OR the IRB approves an assent form that does not include some/all of the required elements. Guidance is available at http://www.research.vcu.edu/irb/wpp/flash/XV-2.htm.

4-C.1. Explain why a waiver or alteration of informed consent is being requested.

In order for the IRB to approve a request for waiver of assent, the conditions for 4-C.2, 4-C.3, <u>OR</u> 4-C.4 must be met. Check which <u>ONE</u> applies and <u>explain</u> all required justifications.

4-C.2. ☐ **Some or all of the individuals age 7 or higher will not be capable of providing assent based on their developmental status or impact of illness**. → Explain how your study meets this criteria:

4-C.3. ☐ **The research holds out a prospect of direct benefit not available outside of the research**. → Explain how your study meets this criteria:

4-C.4. ☐ **Describe how this study meets <u>ALL FOUR</u> of the following conditions**:

- The research involves no more than minimal risk to the participants. → Explain how your study meets this criteria:

- The waiver or alteration will not adversely affect the rights and welfare of participants. → Explain how your study meets this criteria:

- The research could not practicably be carried out without the waiver or alteration. → Explain how your study meets this criteria:

- Will participants be provided with additional pertinent information after participation?
  ☐ Yes
  ☐ No → Explain why not:

4-D. REQUEST TO WAIVE CONSENT FOR EMERGENCY RESEARCH: Describe how the study meets the criteria for

emergency research and the process for obtaining LAR consent is appropriate. See guidance at http://www.research.vcu.edu/irb/wpp/flash/XVII-16.htm.

5. GENETIC TESTING
If applicable, address the following issues related to Genetic Testing.

5-A. FUTURE CONTACT CONCERNING FURTHER GENETIC TESTING RESEARCH
Describe the circumstances under which the subject might be contacted in the future concerning further participation in this or related genetic testing research.

N/A

5-B. FUTURE CONTACT CONCERNING GENETIC TESTING RESULTS
If planned or possible future genetic testing results are unlikely to have clinical implications, then a statement that the results will not be made available to subjects may be appropriate. If results might be of clinical significance, then describe the circumstances and procedures by which subjects would receive results. Describe how subjects might access genetic counseling for assistance in understanding the implications of genetic testing results, and whether this might involve costs to subjects. Investigators should be aware that federal regulations, in general, require that testing results used in clinical management must have been obtained in a CLIA-certified laboratory.

N/A

5-C. WITHDRAWAL OF GENETIC TESTING CONSENT
Describe whether and how subjects might, in the future, request to have test results and/or samples withdrawn in order to prevent further analysis, reporting, and/or testing.

N/A

5-D. GENETIC TESTING INVOLVING CHILDREN OR DECISIONALLY IMPAIRED PARTICIPANTS
Describe procedures, if any, for consenting children upon the attainment of adulthood. Describe procedures, if any, for consenting participants who are no longer decisionally impaired.

N/A

5-E. CONFIDENTIALITY OF GENETIC INFORMATION
Describe the extent to which genetic testing results will remain confidential and special precautions, if any, to protect confidentiality.

N/A
