# Supplementary material for: Enhanced Interleukin-1 Activity Contributes to Exercise Intolerance in Patients with Systolic Heart Failure
Source: PLoS One. 2012 Mar 16;7(3):e33438. doi: 10.1371/journal.pone.0033438 (PMC3306393; doi:10.1371/journal.pone.0033438)
Supplement: Appendix S1 — The Duke Activity Status Index (DASI) questionnaire. (DOCX) [file pone.0033438.s002.docx]

| Appendix 1. The Duke Activity Status Index (DASI) questionnaire |  |
| --- | --- |
| Question | **Weight** |
| 1. Take care of yourself (i.e., eating dressing, bathing, using the toilet)? | 2.75 |
| 2. Walk indoors, such a around your house? | 1.75 |
| 3. Walk a block or two on level ground? | 2.75 |
| 4. Climb a flight of stairs or walk up a hill? | 5.50 |
| 5. Run short distances? | 8.00 |
| 6. Do light work around the house like dusting or washing dishes? | 2.70 |
| 7. Do moderate work around the house like vacuuming, sweeping floors, of carrying in groceries? | 3.50 |
| 8. Do heavy work around the house like scrubbing floors or lifting or moving heavy furniture? | 8.00 |
| 9. Do yard work like raking leaves, weeding, or pushing a power mower? | 4.50 |
| 10. Have sexual relations? | 5.25 |
| 11. Participate in moderate recreational activity such as golf, bowling, dancing, or doubles tennis of throw a basketball or football | 6.00 |
| 12. Participate in strenuous sports such as swimming, singles tennis, football, basketball, or skiing? | 7.50 |

### Calculations

The index equals the sum of weights for “yes” replies

VO_2_ = 0.43 * DASI + 9.6
